# Derivation and external validation of community-acquired pneumonia subphenotypes in Southeast Asia: a secondary analysis of prospective cohort studies

**DOI:** 10.1016/j.eclinm.2025.103572

**Published:** 2025-10-24

**Authors:** Taylor D. Coston, Prapassorn Poolchanuan, Jesse E. Ross, Lu Xia, Leila R. Zelnick, Viriya Hantrakun, Parinya Chamnan, Gumphol Wongsuvan, David Furfaro, Max R. O'Donnell, Ali Shojaie, Sina A. Gharib, Pavan K. Bhatraju, Khie C. Lie, Chuen-Yen Lau, Nguyen V.V. Chau, Matthew J. Cummings, Direk Limmathurotsakul, Shelton W. Wright, T. Eoin West

**Affiliations:** a Division of Pulmonary, Critical Care, and Sleep Medicine, Department of Medicine, University of Washington, Seattle, USA; b Department of Microbiology and Immunology, Faculty of Tropical Medicine, Mahidol University, Bangkok, Thailand; c Division of Pulmonary, Critical Care, and Sleep Medicine, Department of Medicine, David Geffen School of Medicine, University of California, Los Angeles, USA; d Department of Statistics and Probability, Michigan State University, East Lansing, MI, USA; e Division of Nephrology, Department of Medicine, University of Washington, Seattle, USA; f Mahidol Oxford Tropical Medicine Research Unit, Faculty of Tropical Medicine, Mahidol University, Bangkok, Thailand; g Department of Social Medicine, Sunpasitthiprasong Hospital, Ubon Ratchathani, Thailand; h Division of Pulmonary, Allergy, and Critical Care Medicine, Beth Israel Deaconess Medical Center, Brookline, MA, USA; i Division of Pulmonary, Allergy, and Critical Care Medicine, Department of Medicine, Vagelos College of Physicians and Surgeons, Columbia University, New York, USA; j Center for Infection and Immunity, Mailman School of Public Health, Columbia University, New York, USA; k Department of Epidemiology, Mailman School of Public Health, Columbia University, New York, NY, USA; m Department of Biostatistics, University of Washington, Seattle, USA; n Division of Tropical and Infectious Diseases, Department of Internal Medicine, Faculty of Medicine Universitas Indonesia, Dr. Cipto Mangunkusumo National General Hospital, Jakarta, Indonesia; o HIV Dynamics and Replication Program, Center for Cancer Research, National Cancer Institute, National Institutes of Health, Bethesda, MD, USA; p Oxford University Clinical Research Unit, Hospital for Tropical Diseases, Ho Chi Minh City, Viet Nam; q Department of Tropical Hygiene, Faculty of Tropical Medicine, Mahidol University, Bangkok, Thailand; r Division of Pediatric Critical Care Medicine, Department of Pediatrics, University of Washington, Seattle, USA; s Department of Global Health, University of Washington, Seattle, USA

**Keywords:** Pneumonia, Precision medicine, Metabolomics, Low- and middle-income countries, Subphenotypes

## Abstract

**Background:**

Identifying pneumonia subphenotypes in understudied populations can advance equitable personalized medicine for pneumonia care. We aimed to derive and validate subphenotypes of patients presenting with community-acquired pneumonia (CAP) in Southeast Asia.

**Methods:**

This secondary analysis included three prospective cohorts conducted between March 2013 and April 2020. First, we performed latent class analysis to identify subphenotypes using clinical and laboratory variables in a prospective cohort of adults hospitalized with CAP in northeastern Thailand. Next, we compared clinical and biological features between the subphenotypes and then developed a parsimonious classifier model (PCM) for accurate subphenotype assignment. We then validated the accuracy of PCM subphenotype assignment in an external, multinational prospective cohort of patients hospitalized with CAP in Southeast Asia. Finally, in an exploratory analysis, we used the PCM to assign pneumonia subphenotypes in a prospective cohort of patients hospitalized with COVID-19 in the United States of America and evaluated a heterogeneity of treatment effect with corticosteroids.

**Findings:**

Among 953 CAP patients in the Thai derivation cohort, we identified two subphenotypes: CAP1 (141, 15%) and CAP2 (812, 85%). We observed greater respiratory failure, 28-day mortality, inflammatory cytokines and metabolic derangements among CAP1 patients. A four-variable PCM discriminated subphenotype assignment in bootstrap internal validation (optimism-corrected C-statistic 0.97). In the Southeast Asian external validation cohort, CAP1 and CAP2 subphenotypes assigned by the PCM shared similar differential clinical features and outcomes with the Thai derivation cohort. In the cohort of patients with COVID-19, CAP1 and CAP2 subphenotypes assigned by the PCM differed by key clinical characteristics and revealed an interaction between corticosteroid treatment and mortality (P = 0.002).

**Interpretation:**

In Southeast Asia, CAP subphenotypes are associated with distinct outcomes, inflammatory profiles, and metabolomic signatures. These subphenotypes may represent unique targets for future CAP interventional trials.

**Funding:**

Supported by US NIH awards T32HL007287, F32HL168809, K08HL157562, U01AI115520, R01AI137111, R01GM114029, R21AI173435, R01HL113382, the 10.13039/100010269Wellcome Trust grants 090219/Z/09/Z, 101103/Z/13/Z, 106680/B/14/Z, and 106698/B/14/Z, the US National Cancer Institute, 10.13039/100000002National Institutes of Health HHSN261200800001E, and 10.13039/100005815Firland Foundation award 20220012. For the purpose of Open Access, the author has applied a CC BY public copyright license to any Author Accepted Manuscript version arising from this submission.

**Research in context**

**Evidence before this study**

Most studies investigating subphenotypes or endotypes in sepsis patients have been performed in high-income countries (HICs) with few investigating community-acquired pneumonia (CAP), a leading infectious cause of death globally. We searched PubMed for studies published up to July 2024 using the search terms: (“community-acquired pneumonia” OR “CAP”) AND (“subphenotypes” OR “endotypes” OR “latent class analysis” OR “unsupervised clustering”) with no language restrictions and identified 35 studies. Of these, five studies investigated CAP subphenotypes or endotypes, though none were performed in low- or middle-income countries, all included variables that may not be widely available in resource-limited settings, and only two included external validation.

**Added value of this study**

To our knowledge, this is the first study to identify and validate subphenotypes of CAP in a resource-limited setting and the first metabolomic comparison of CAP subphenotypes. By applying latent class analysis to clinical variables in a prospective cohort in Southeast Asia, we identified two CAP subphenotypes that differed in inflammatory profiles, metabolomic signatures, and outcome. A parsimonious model of four routinely available clinical and laboratory variables discriminated subphenotype assignment in a multinational external validation cohort in Southeast Asia. Subphenotypes were also reproduced in a COVID-19 cohort in the U.S. and revealed an interaction between corticosteroid treatment and mortality. The strengths of this study include its large derivation cohort and multicenter external validation cohort from multiple countries in Southeast Asia. Furthermore, our dataset featured limited missing data, near-universal follow-up, and analyzed only variables from within the first 24 h after admission to the study hospital.

**Implications of all the available evidence**

We identified CAP subphenotypes derived in a unique, tropical setting defined by distinct inflammatory profiles, metabolomic signatures, and outcome. The ability to classify CAP patients using a simple four-variable model could facilitate rapid bedside risk stratification and enable future studies to test differences in treatment responses by the subphenotypes. As most subphenotype studies are performed in HICs, this study represents an important step toward context-specific precision medicine.

## Introduction

Recent advancements in translational critical care have highlighted shortcomings in the prevailing syndrome-based approach to managing critical illness.^1^^,^^2^ Investigations into host responses have delineated heterogeneous subgroups, termed subphenotypes, in patients with sepsis, acute kidney injury, and acute respiratory distress syndrome (ARDS), some of which differ in their response to certain interventions.^3^, ^4^, ^5^, ^6^, ^7^, ^8^ Consequently, there is an increasing focus on implementing a precision medicine approach, where future management strategies could be based on subphenotype identification. This approach holds potential for improving outcomes in acute care settings.^1^

The vast majority of studies investigating subphenotypes in infected patients have been performed in high-income countries (HICs).^3^^,^^4^^,^^9^^,^^10^ Few studies have investigated community-acquired pneumonia (CAP) specifically, which may exhibit distinct host immune responses compared with other infectious etiologies.^7^^,^^11^, ^12^, ^13^ Pneumonia, the leading infectious cause of death globally, is associated with 2.5 million deaths yearly.^14^ Low- and middle-income countries (LMICs), including in Southeast Asia, suffer the highest pneumonia mortality rates.^14^ Patients with CAP in tropical regions of Southeast Asia exhibit distinct demographics, comorbidities, and etiologic pathogens compared to those in HICs.^15^^,^^16^ Even well-established interventions for severe infection in HICs have proven ineffective or detrimental in LMICs, including sepsis protocols in sub-Saharan Africa.^17^^,^^18^ Such heterogeneity of treatment effect across populations may be due, in part, to yet unrecognized subphenotypes reflecting setting-specific factors.^19^ Infection-related subphenotypes have limited reproducibility, therefore, contextualized research is necessary in regions most burdened by CAP.^20^

We hypothesized that commonly available clinical and laboratory data could be used to categorize patients with CAP into distinct subphenotypes in a prospective cohort of patients with community-acquired infection in northeastern Thailand. We then sought to externally validate these subphenotypes in a multinational multicenter cohort in SE Asia. Finally, to explore whether subphenotype outcomes may differ by treatment, we sought to identify these subphenotypes in patients hospitalized with COVID-19 in the United States and assessed for a differential relationship between corticosteroid treatment and mortality.^21^, ^22^, ^23^, ^24^

## Methods

### Study participants

#### Derivation cohort

**Fig. 1:**
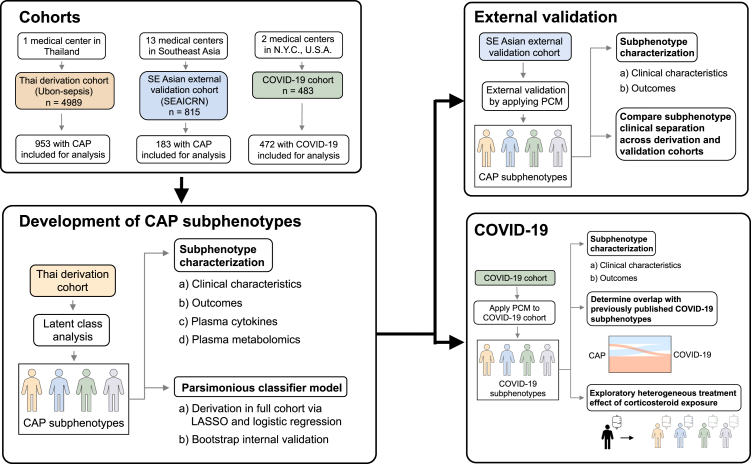
Study flow diagram. *Abbreviations*: CAP = community-acquired pneumonia, LASSO = Least Absolute Shrinkage and Selection Operator, N.Y.C. = New York City, U.S.A. = United States of America, PCM = parsimonious classifier model.

Patients aged 18 years or older admitted to Sunpasitthiprasong Hospital in Ubon Ratchathani, Thailand, with suspected infection were prospectively enrolled into the Thai derivation cohort between 2013 through 2017 (Ubon-sepsis, Fig. 1).^23^ This cohort has been described previously.^23^ Enrollment occurred if patients had been admitted within the previous 24 h with attending physician suspicion of infection and at least three documented systemic manifestations of infection, according to the 2012 Surviving Sepsis Campaign.^25^ This secondary analysis focused on patients with CAP defined by the presence of all three of the following criteria: 1) respiratory symptoms, 2) primary admission diagnosis of pneumonia by the treating clinician, and 3) final (discharge) diagnosis of pneumonia or melioidosis. Discharge diagnosis of melioidosis was included in the CAP criteria because it is common practice to record a melioidosis pneumonia patient as only “melioidosis” in the medical record.

#### External validation cohort

Patients aged 18 years or older admitted with suspected infection at 13 referral hospitals in three countries in Southeast Asia (Thailand, Vietnam, and Indonesia) were prospectively enrolled into the SE Asian external validation cohort between 2013 and 2015 (SEAICRN, Fig. 1); this cohort has been described previously and utilized the same enrollment criteria for adult participants as Ubon-sepsis.^24^ CAP criteria were similar to those used in Ubon-sepsis and are described in the Supplementary Material.

#### COVID-19 cohort

A third cohort of mechanically ventilated adults with COVID-19 in the U.S. was analyzed to assess if subphenotypes could be reproduced.^7^^,^^26^ Among patients age 18 years or older admitted to intensive care units at two hospitals affiliated with New York-Presbyterian Hospital/Columbia University Irving Medical Center from March 2, 2020, through April 30, 2020, patients were included in the COVID-19 cohort if they fulfilled the following criteria: 1) had a positive PCR test for SARS-CoV-2, 2) required invasive mechanical ventilation, and 3) met criteria for ARDS using the Berlin definition.^27^ Clinical and laboratory variables were recorded on the initial day of mechanical ventilation unless otherwise specified.

### Latent class analysis

Baseline demographic data, clinical data, and routine laboratory tests were considered as class-defining variables in the latent class analysis (LCA) model; classification was conducted in the Thai derivation cohort without consideration of clinical outcomes. The list of 25 class-defining variables was decided *a priori* based on literature supporting their role in CAP, their inclusion in published subphenotyping studies in sepsis and ARDS, and by missingness and correlation criteria according to best practices as described in the Supplementary Material and Supplementary Tables S1 and S2.^5^^,^^11^^,^^28^^,^^29^ All clinical variables were assessed at screening within 24 h of admission to the study hospital, and laboratory variables were obtained on day zero or day one after admission to the study hospital. Variables were processed prior to LCA and model fit determined as described in the Supplementary Material.^29^ LCA was conducted using Mplus version 8.9 (Muthen and Muthen, Los Angeles, CA).

### Outcomes

The association of CAP subphenotype with 28-day mortality unadjusted and adjusted for age, sex, comorbidities, modified Sequential Organ Failure Assessment (SOFA) score, and outside hospital transfer status was evaluated using the Cox proportional hazards model. Statistical analyses other than LCA were performed using Stata/SE version 14.2 (College Station, TX).

### Plasma cytokine assays in the derivation cohort

Plasma samples were obtained at the time of enrollment from all patients in the parent cohort and frozen at −80 °C.^23^ Cytokine assays were performed on plasma from a subset of subjects randomly selected from each subphenotype. IL-6, TNF-α, IL-1β, and IL-10, chosen based on their role in CAP host responses, were measured by electrochemiluminescence multiplex assay (Meso Scale Discovery, Rockville, MD) and were log_2_ transformed for analysis. Linear regression was performed separately for each cytokine with CAP subphenotype as the independent variable adjusted by modified SOFA score and transfer status.^30^, ^31^, ^32^

### Metabolomic analyses in the derivation cohort

Plasma metabolomic profiles were analyzed in a CAP subset in the Thai derivation cohort with metabolomic data available from a separate study, in which subjects were systematically selected by pathogen as described in the Supplementary Material.^33^ In brief, plasma was obtained at enrollment from all patients in Ubon-sepsis. Subjects with a positive culture for *Burkholderia pseudomallei* were selected for plasma metabolomics as well as a random sample from those who were bacteremic due to other pathogens, and a random sample from those who were culture negative. Sample preparation and ultrahigh performance liquid chromatography-tandem mass spectrometry of plasma samples were performed by Metabolon, Inc. (Morrisville, NC, USA).^33^ Linear regression was performed with log_2_ metabolite abundance as the dependent variable and CAP subphenotype as the independent variable, and the R package “globaltest” (version 5.50.0) was used for pathway analysis.

### Development of a classifier and external validation of subphenotypes

A parsimonious classifier model (PCM) to identify subphenotype assignment was derived, internally validated, and applied to external cohorts as described in the Supplementary Material. In brief, Least Absolute Shrinkage and Selection Operator (LASSO), a regression technique that retains only the most important variables needed for prediction, identified the fewest variables predictive of subphenotype assignment.^34^ Logistic regression using the LASSO-identified variables was then used to develop a PCM in the full Thai derivation cohort. The optimal cut-point for subphenotype assignment was calculated using the Youden index method. Then, internal validation was performed via bootstrap validation with 200 replicates to obtain an optimism-corrected C-statistic, as recommended by Harrell et al.^35^, ^36^, ^37^ The PCM was then applied to the SE Asian external validation cohort to classify subphenotypes. Proportion of subphenotypes, clinical variables, and outcomes were compared between subphenotypes in the external validation cohort in the same manner as in the derivation cohort.

### Evaluation of subphenotypes in COVID-19

Next, the PCM and optimal cut-point were applied to an external cohort of adults hospitalized with COVID-19 receiving mechanical ventilation in NYC, U.S.A. to assign subphenotypes.^26^ Clinical and biological characteristics were compared between assigned subphenotypes and mortality was assessed in unadjusted and adjusted analyses. Overlap between these CAP subphenotypes derived from the Thai derivation cohort and previously published COVID-19 subphenotypes was reported by Cohen's kappa. Cox regression was performed with an interaction term between subphenotype assignment and corticosteroid exposure to assess if an association between corticosteroid exposure (non-randomized) and death differed by CAP subphenotype.

### Ethics

Written informed consent was obtained from study participants or their representatives prior to enrollment in the parent Ubon-sepsis study and SEAICRN study, and waiver of consent was applied for the COVID-19 cohort. These studies were approved by the Sunpasitthiprasong Hospital Ethics Committee (039/2556), the Ethics Committee of the Faculty of Tropical Medicine, Mahidol University (MUTM2012-024-01 and MUTM2024-022-01), the University of Washington Institutional Review Board (42988), the Oxford University Tropical Research Ethics Committee (OXTREC172-12 and 530-24), appropriate local and national ethics committees in SE Asia, and the institutional review board at Columbia University Irving Medical Center (AAAS8916).

### Role of the funding source

The funding source had no role in study design, data collection, analysis, decision to publish, nor preparation of the manuscript.

## Results

### Patient characteristics

Of the 4989 subjects enrolled into the parent Ubon-sepsis cohort, 953 met criteria for CAP and were included in the Thai derivation cohort (Table 1). The median age was 67 years (interquartile range [IQR] 52–78) and 413 (43%) were female. Median modified SOFA score was 4 (IQR 2–6) and 28-day mortality was 291/953 (30.5%).

**Table 1:** Characteristics of the derivation, external validation, and COVID-19 cohorts.

Characteristics^a^	Thai derivation cohort (Ubon-sepsis)	SE Asian external validation cohort (SEAICRN)	COVID-19 cohort
**No. of patients**	953	183	472
**Demographics**			
Age in years, median (IQR)	67 (52–78)	59 (45–75)	65 (55–73)
Female sex, n (%)	413 (43.3)	70 (38.3)	157 (33.3)
**Pre-existing conditions, n (%)**			
Diabetes	220 (23.1)	33 (18.0)	205 (43.4)
Chronic kidney disease	124 (13.0)	13 (7.1)	–
Chronic cardiovascular disease	104 (10.9)	11 (6.0)	116 (24.6)
Chronic lung disease	176 (18.5)	13 (7.1)	–
Cancer	25 (2.6)	3 (1.6)	–
HIV	14 (1.5)	2 (1.1)	–
**Transferred from another facility, n (%)**	802 (84.2)	69 (37.7)	–
**SOFA score, median (IQR)**^b^	4 (3–7)	2 (0–5)	7 (6–9)
**28-day mortality, n (%)**	291 (30.5)	48 (26.5)	252 (53.6)

*Abbreviations*: HIV = Human Immunodeficiency Virus; SOFA = Sequential Organ Failure Assessment.

a Continuous variables are presented as median (interquartile range) and categorical data are presented as n (%).

b Modified SOFA score presented for Ubon-sepsis and SEAICRN is described in the Supplementary Material.

### Clinical features of subphenotypes in the derivation cohort

**Fig. 2:**
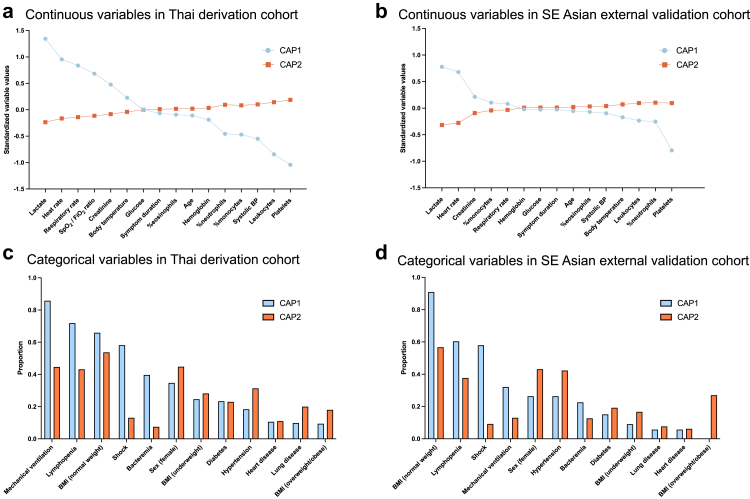
Clinical characteristics and outcomes by subphenotype in the Thai derivation cohort and SE Asian external validation cohort. (*a*) Continuous variables (standardized) by subphenotype assignment in Thai derivation cohort and (*b*) SE Asian external validation cohort. Differences between the standardized values of each variable by subphenotype (y-axis) for the variable shown on the x-axis. The variables are sorted by degree of separation between subphenotypes: from the maximum positive separation on the left (where the standardized value of CAP1 is higher than the standardized value of CAP2) to the maximum negative separation on the right (where the standardized value of CAP1 is lower than the standardized value of CAP2). The crossover of the lines indicates that the standardized value for this variable was the same for CAP1 and CAP2 (i.e., no difference between CAP1 or CAP2 for this variable). Therefore, variables near the intersection of both lines are similar in both subphenotypes. In the Thai derivation cohort, SpO 2/FiO2 ratio is on an ordinal scale as described in the Supplementary Material. (*c*) Categorical variable proportions by subphenotype assignment in the Thai derivation cohort and (*d*) SE Asian external validation cohort. Lymphopenia was defined as absolute lymphocyte count <1.0 × 10^9^/L. *Abbreviations*: BMI = body mass index, BP = blood pressure.

LCA was applied to the Thai derivation cohort and model fit characteristics are summarized in Supplementary Table S3. A two-class model provided the best fit and was chosen for further analysis with subphenotypes termed CAP1 and CAP2. Out of 953 total patients, 15% were assigned to CAP1 (141/953) and 85% were assigned to CAP2 (812/953). Several clinical features differentiated CAP1 from CAP2 as shown by the standardized mean differences in Table 2 and Fig. 2. Compared with CAP2, patients assigned to CAP1 had higher modified SOFA score, more abnormal vital signs, and higher proportion of patients receiving vasopressors and/or mechanical ventilation (Table 2, Fig. 2). Age, symptom duration, body temperature, and proportion of patients with diabetes were all similar between the subphenotypes. Patients assigned to CAP1 also had more frequent bacteremia (Table 2 and Supplementary Table S4). Among bacteremic patients, the Gram-negative pathogens *B. pseudomallei* and *Klebsiella pneumoniae* were more frequent in CAP1, while the Gram-positive pathogen *Streptococcus pneumoniae* was more frequent in CAP2.

**Table 2:** Baseline variables and outcome by subphenotypes in the derivation and external validation cohorts.

	Thai derivation cohort (Ubon-sepsis)			SE Asian external validation cohort (SEAICRN)		
	CAP1	CAP2	Difference (95% CI)^a^	CAP1	CAP2	Difference (95% CI)
**Subjects**	141	812	–	53	130	–
**Baseline demographics**						
Age in years	64 (50–75)	68 (53–78)	4.0 (−9.0 to 1.0)	57 (46–69)	60 (45–78)	−3.0 (−10.0 to 5.0)
Female sex	49 (34.8)	364 (44.8)	−10.1 (−18.4 to −1.3)	14 (26.4)	56 (43.1)	−16.7 (−31.1 to 1.5)
Body mass index, kg/m^2^						
<18.5	34 (24.6)	224 (28.2)	−3.6 (−11.3 to 4.5)	1 (9.1)	10 (16.7)	−7.6 (−23.7 to 15.1)
18.5–24.9	91 (65.9)	426 (53.7)	12.2 (3.5–20.8)	10 (90.9)	34 (56.7)	34.2 (9.4–53.1)
25–29.9	11 (8.0)	107 (13.5)	−5.5 (−10.4 to −0.2)	0 (0.0)	11 (18.3)	−18.3 (−28.6 to −8.9)
≥30	2 (1.4)	36 (4.5)	−3.1 (−5.3 to −0.3)	0 (0.0)	5 (8.3)	−8.3 (−15.7 to −1.8)
**Comorbidities**						
Diabetes	33 (23.4)	187 (23.0)	0.4 (−7.0 to 8.0)	8 (15.1)	25 (19.2)	−4.1 (−15.6 to 8.0)
Heart disease	15 (10.6)	89 (11.0)	−0.3 (−5.6 to 5.4)	3 (5.7)	8 (6.2)	−0.5 (−7.7 to 7.5)
Chronic lung disease	14 (9.9)	162 (20.0)	−10.0 (−15.4 to −4.3)	3 (5.7)	10 (7.7)	−2.0 (−9.7 to 5.9)
Smoking	4 (2.8)	17 (2.1)	0.7 (−1.9 to 3.9)	7 (13.2)	20 (15.4)	−2.2 (−12.8 to 9.2)
Hypertension	26 (18.4)	255 (31.4)	−13.0 (−20.0 to −5.5)	14 (26.4)	55 (42.3)	−15.9 (−29.9 to −1.1)
Chronic kidney disease	12 (8.5)	112 (13.8)	−5.3 (−10.2 to 0.1)	4 (7.5)	9 (6.9)	−0.6 (−7.3 to 9.4)
Cancer	3 (2.1)	22 (2.7)	−0.6 (−2.3 to 1.0)	0 (0)	3 (2.3)	−2.3 (−5.4 to 0.0)
HIV	1 (0.7)	13 (1.6)	−0.8 (−0.9 to 1.0)	0 (0)	2 (1.5)	−1.5 (−3.8 to 0.0)
**Symptoms**						
Symptom duration, days						
≤2 days	61 (43.3)	323 (39.8)	3.5 (−5.3 to 12.3)	18 (34.0)	56 (43.1)	−9.1 (−23.9 to 6.1)
3–7 days	67 (47.5)	407 (50.1)	−2.6 (−11.5 to 6.6)	32 (60.4)	60 (46.2)	14.2 (−1.4 to 29.4)
>7 days	13 (9.2)	82 (10.1)	−0.9 (−5.9 to 4.6)	3 (5.7)	14 (10.8)	−5.1 (−12.7 to 3.6)
**Vital signs**						
Body temperature, °C	38 (37–38)	37 (36–38)	0.4 (−0.1 to 0.7)	38 (37–39)	38 (37–39)	−0.4 (−1.2 to 0.2)
Heart rate, beats per minute	110 (100–124)	92 (82–102)	18.0 (16.0–22.0)	116 (102–130)	98 (85–110)	18.0 (12.0–29.0)
Respiratory rate, breaths per minute	24 (20–28)	20 (20–24)	4.0 (4.0–6.0)	24 (20–32)	24 (20–30)	0.5 (−4.0 to 4.0)
Systolic blood pressure, mmHg	100 (90–120)	119 (102–134)	−19.0 (−21.0 to −9.0)	118 (100–135)	120 (104–140)	−2.0 (−14.0 to 7.0)
**Organ failure/life support**						
Mechanical ventilation	121 (85.8)	363 (44.7)	41.1 (34.3–47.8)	17 (32.1)	17 (13.1)	19.0 (5.4–33.3)
Modified SOFA score	9 (6–11)	3 (1–5)	6.0 (5.0–6.0)	6 (4–10)	2 (0–5)	5.0 (3.0–7.0)
SpO_2_/FiO_2_ ordinal score	3 (2–4)	2 (0–2)	1.0 (1.0–1.0)	–	–	–
CURB-65	2 (1–3)	1 (1–2)	1.0 (0.0–2.0)	2 (1–3)	2 (1–2)	0.0 (0.0–1.0)
Vasopressors	82 (58.2)	106 (13.1)	45.1 (36.7–53.4)	31 (58.5)	12 (9.2)	49.3 (34.9–63.3)
**Routine laboratory biomarkers**						
Leukocyte count, x 10^−9^/L	7.2 (3.2–16.5)	13.5 (10.0–18.7)	−6.3 (−8.1 to −3.6)	9 (6–16)	15 (9–19)	−5.2 (−7.1 to −2.0)
%neutrophils	83 (68–91)	85 (79–90)	−2.0 (−6.0 to 0.0)	83 (75–90)	83 (77–89)	−0.1 (−3.8 to 5.2)
%monocytes	3 (1–5)	5 (3–7)	−2.0 (−3.0 to −1.0)	4 (2–6)	5 (3–7)	−1.0 (−2.2 to 0.1)
%eosinophils	0 (0–1)	0 (0–1)	0.0 (0.0–0.0)	0 (0–0)	0 (0–1)	−0.2 (−0.3 to −0.1)
%lymphocytes	9 (5–20)	8 (5–13)	1.0 (1.0–4.0)	9 (5–15)	10 (6–16)	−0.9 (−3.4 to 2.0)
%basophils	0 (0–0)	0 (0–0)	0.0 (0.0–0.0)	0 (0–0)	0 (0–0)	−0.2 (−0.3 to −0.1)
Lymphopenia, <1.0 × 10^9^/L	100 (71.9)	339 (43.2)	28.8 (20.3–36.9)	32 (60.4)	49 (37.7)	22.7 (6.7–38.6)
Hemoglobin, g/dL	10 (9–12)	11 (9–12)	−0.6 (−0.8 to 0.0)	12 (10–14)	12 (11–14)	−0.3 (−1.6 to 0.8)
Platelet count, x 10^−9^/L	118 (58–169)	232 (168–314)	−114.0 (−142.0 to −97.0)	111 (40–181)	242 (180–307)	−131.5 (−172.0 to −88.0)
Creatinine, mg/dL	1.9 (1.3–3.3)	1.2 (0.8–1.9)	0.7 (0.6–1.1)	2 (1–4)	1 (1–2)	0.7 (0.3–1.4)
Lactate, mmol/L	5.4 (3.0–9.5)	1.8 (1.3–2.6)	3.6 (2.8–4.5)	5 (2–9)	2 (1–2)	2.9 (1.3–4.1)
Blood glucose, mg/dL	154 (108–207)	137 (111–181)	17.0 (−4.0 to 31.0)	139 (109–170)	128 (110–174)	10.5 (−14.5 to 26.5)
**Micro data**						
Bacteremia	56 (39.7)	61 (7.5)	32.2 (24.0–40.5)	12 (22.6)	16 (12.7)	9.9 (−2.4 to 23.2)
**Transferred from outside facility**	130 (92.2)	672 (82.8)	9.4 (4.1–14.3)	31 (58.5)	38 (29.2)	29.3 (13.4–44.5)

	Thai derivation cohort (Ubon-sepsis)				SE Asian external validation cohort (SEAICRN)			
	CAP1	CAP2	HR (95% CI)	P-value	CAP1	CAP2	HR (95% CI)	P-value
Death within 28 days	100/141 (70.9)	191/812 (23.5)	–	–	28/53 (54.9)	20/130 (15.4)	–	–
Unadjusted			4.81 (3.77–6.13)	<0.001			4.14 (2.38–7.18)	<0.001
Adjusted^b^			2.60 (1.90–3.56)	<0.001			2.25 (0.97–5.18)	0.058

*Abbreviations*: HIV = Human Immunodeficiency Virus; SOFA = Sequential Organ Failure Assessment.

Continuous variables are presented as median (interquartile range) and categorical data are presented as n (%).

a Difference in medians or proportions with 95% confidence intervals (CIs) estimated over 10,000 bootstrapped replicates.

b Hazard ratios were calculated with the use of Cox regression, unadjusted and adjusted for age, sex, modified SOFA score, transfer status, and comorbidities (derivation cohort adjusted for Charlson Comorbidity Index; external validation cohort adjusted for individual binary variables diabetes, lung disease, kidney disease, alcohol use, smoking).

### CAP subphenotypes and death

**Fig. 3:**
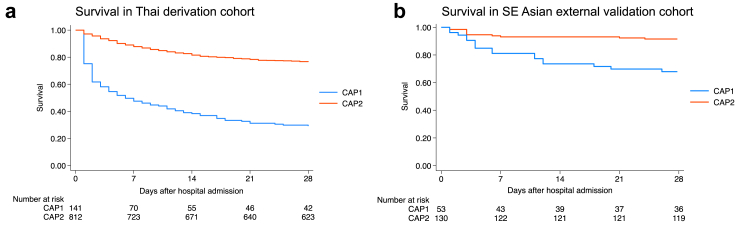
Kaplan–Meier survival curves by subphenotype in the (*a*) Thai derivation cohort and (*b*) SE Asian external validation cohort.

One-hundred of 141 (71%) patients assigned to CAP1 died by 28 days, and 191/812 (24%) of patients assigned to CAP2 died by 28 days. In unadjusted Cox regression, CAP1 assignment was associated with higher hazard of death within 28-days compared with CAP2 (71% vs. 24%, HR 4.8, 95% CI 3.8–6.1, P < 0.001; Table 2, Fig. 3). Given the evident differences in illness severity between CAP1 and CAP2, we assessed if CAP1 was associated with death adjusted for relevant indicators of severity. Compared with CAP2, CAP1 remained associated with higher hazard of death in Cox regression adjusted for age, sex, Charlson Comorbidity Index, modified SOFA score, and transfer status (HR 2.60, 95% CI 1.90–3.56, P < 0.001; Table 2).

### CAP subphenotypes and biological profiles

**Fig. 4:**
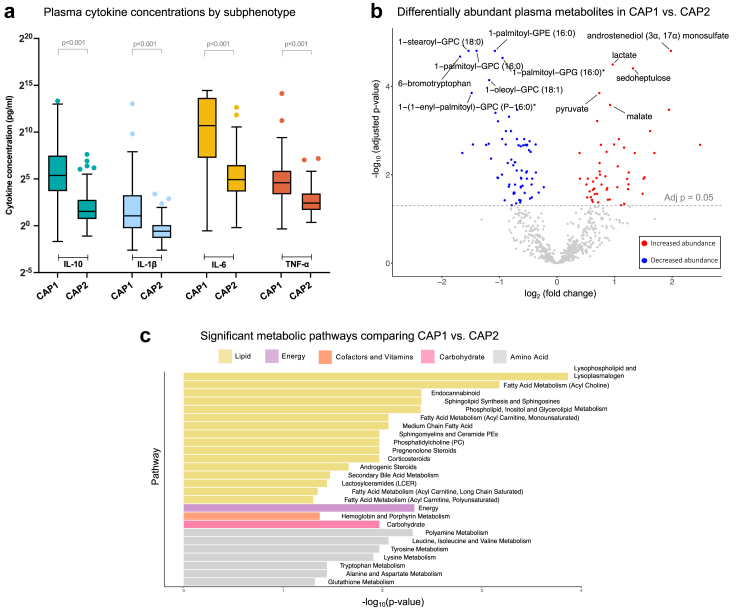
Biological comparisons between subphenotypes in the Thai derivation cohort: (*a*) Plasma cytokine concentrations by subphenotype. (*b*) Volcano plot comparing differential abundance of plasma metabolites between CAP1 and CAP2 adjusted for age, sex, and modified SOFA score. Significant metabolites after Benjamini-Hochberg P-value adjustments are highlighted; red: increased abundance, blue: decreased abundance. (*c*) Barplot of 26 significant metabolic pathways obtained from comparing CAP1 with CAP2 adjusted for age, sex, and modified SOFA score. Height of the bars represents negative log_10_ of Benjamini-Hochberg procedure adjusted P-values. *Abbreviations*: CAP = community-acquired pneumonia, SOFA = Sequential Organ Failure Assessment.

We then compared plasma inflammatory cytokine concentrations at the time of enrollment between subphenotypes. Patients assigned to CAP1 had markedly higher enrollment concentrations of IL-6, TNF-α, IL-1β, and IL-10 compared to CAP2 (Supplementary Table S5, Fig. 4A). Next, we performed metabolomic analyses to investigate the under-studied role of the host metabolic response to infection, which may contribute to heterogeneity in CAP.^9^^,^^38^, ^39^, ^40^ We analyzed the 125 subjects (CAP1: n = 50, CAP2: n = 75) with enrollment timepoint plasma metabolomic data available in the derivation cohort (782 metabolites). Baseline characteristics were similar in this subset compared with the full Ubon-sepsis CAP cohort (Supplementary Table S7) and etiologies of bacteremia showed similar separation between CAP1 and CAP2 in this subset (Supplementary Table S8).

In the models adjusted for modified SOFA score, 123 plasma metabolites were significantly differentially abundant comparing subphenotypes (Supplementary Tables S9 and S10, adjusted P < 0.05; Fig. 4B). Compared to those assigned to CAP2, CAP1 patients had increased metabolites associated with energy superpathways including glycolysis, and decreased metabolites associated with lipid superpathways including lysophospholipids. Out of 67 investigated pathways, 26 were significantly different by subphenotype (adjusted P < 0.05, Supplementary Table S11, Fig. 4C) and were consistent with the individual metabolite analyses.

### Development of a classifier model to identify CAP subphenotypes

We next sought to develop a parsimonious classifier model (PCM) to accurately classify patients by subphenotype. A four-variable model was identified using LASSO to classify subphenotype in the Thai derivation cohort: venous lactate, platelet count, vasopressor use [yes/no], and heart rate. When tested via bootstrap internal validation, this four-variable PCM gave an optimism-corrected C-statistic of 0.97 (95% CI 0.96–0.99, Supplementary Table S12) to discriminate subphenotype assignment. When compared with modified SOFA scores (0.89, 95% CI 0.87–0.93) and CURB-65 (0.68, 95% CI 0.62–0.73), the four-variable PCM had superior subphenotype discrimination (Supplementary Table S12).

### External validation of CAP subphenotypes in SE Asia

We next attempted to validate the subphenotypes by applying the PCM to an external cohort (SEAICRN), which utilized the same criteria as the Thai derivation cohort. Of the 815 adults enrolled in the SEAICRN cohort, 185 met CAP criteria; two were missing at least one PCM variable and were excluded. Therefore, 183 patients were analyzed in the SE Asian external validation cohort. The median age was 59 years (IQR 45–75) and 70 (38%) were female. Median modified SOFA score was 2 (IQR 0–5) and 28-day mortality was 48/183 (27%).

The PCM was applied to the SE Asian external validation cohort: 53/183 (29%) were assigned to CAP1 and 130/183 (71%) to CAP2. Separation of clinical variables between CAP1 and CAP2 (Fig. 2B and D) was similar to separation observed in the Thai derivation cohort, and standardized mean differences between CAP1 and CAP2 strongly correlated in the derivation and external validation cohorts (Pearson r = 0.82; 95% CI 0.61–0.92; P < 0.001; Supplementary Figure S3). Consistent with the derivation cohort, patients assigned to CAP1 vs. CAP2 in the external validation cohort exhibited a more severe clinical phenotype including higher modified SOFA score, more abnormal vital signs, and higher proportion of patients receiving vasopressors and/or mechanical ventilation (Table 2). As in Ubon-sepsis, CAP1 had lower total leukocyte count and higher proportion of patients with lymphopenia and bacteremia. Pathogen differences were similar to the derivation cohort as well, though the smaller sample size in external validation yielded few positive blood cultures for each pathogen. Finally, paralleling the Thai derivation cohort, patients assigned to CAP1 had higher hazard of death compared with those assigned to CAP2 (Table 2, Fig. 3) and the HRs for death in the external validation cohort (crude HR 4.14, 95% CI 2.38–7.18, adjusted HR 2.25, 95% CI 0.97–5.18) were similar to the HRs in the derivation cohort (Table 2).

### Evaluation of subphenotypes in COVID-19 in the U.S.

We hypothesized that the subphenotypes identified in patients with pneumonia in SE Asia may be shared by cohorts of patients in other settings and with different causes of pneumonia. We applied the four-variable PCM and optimal cut-point to a cohort of adults with COVID-19 receiving mechanical ventilation in the U.S. Of 483 subjects in the cohort, 472 had no missingness in PCM variables and were included for analysis. The median age was 65 (IQR 55–73) and 157 (32%) were female, median SOFA score was 7 (6–9), and 90-day mortality was 252/472 (54%). Two hundred eight of 472 (44%) patients were classified as CAP1 and 264/472 (56%) patients were classified as CAP2 (Supplementary Table S13). Clinical characteristics are shown in Supplementary Table S14, with CAP1 again exhibiting higher markers of inflammation and illness severity compared with CAP2. CAP1 and CAP2 exhibited moderate overlap with inflammatory subphenotypes previously derived in the COVID-19 cohort (CARDS class 1 and CARDS class 2, Cohen's kappa = 0.42, Supplementary Figure S4).^7^ Notable differences compared with the CAP subphenotypes in SE Asia included similar total leukocyte count and similar proportion of lymphopenia between CAP1 and CAP2, and the adjusted hazard ratio of death comparing CAP1 vs. CAP2 (HR 1.22; 95% CI 0.92–1.61) was lower than that observed in the SE Asia cohort. Overall, the analysis in the COVID-19 cohort produced subphenotypes that shared many, but not all, characteristics compared with the SE Asia CAP subphenotypes and moderately overlapped with a subphenotyping schema developed in a high-income country setting.

### Differential response to corticosteroids in COVID-19

**Fig. 5:**
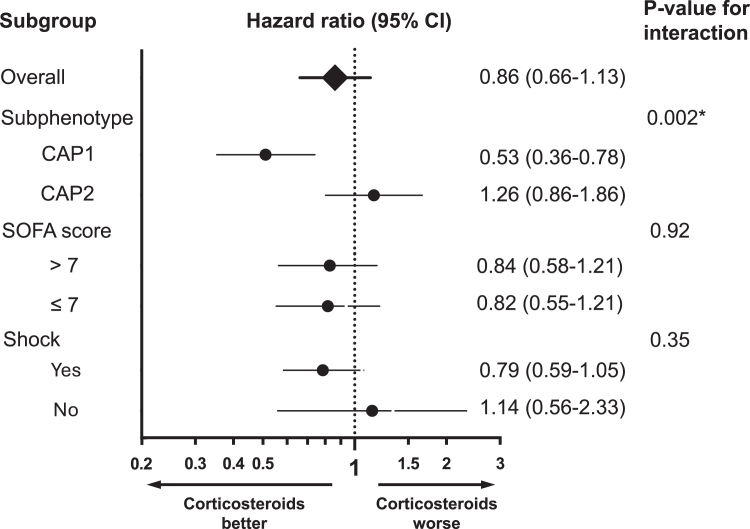
Results of applying the four-variable parsimonious classifier model (lactate, platelet count, vasopressor use, heart rate) to a cohort of 472 adults with COVID-19 receiving mechanical ventilation at two centers in New York City, U.S.A. The hazard ratio and 95% confidence interval are shown overall and according to subgroup for 90-day mortality. The P-value for the steroid exposure and subphenotype interaction term in unadjusted Cox regression is presented. SOFA score is dichotomized at the median for the COVID-19 cohort and shock defined as receipt of vasopressors.

Because of the differences in inflammatory markers between the subphenotypes, we hypothesized that patients with the CAP1 subphenotype may benefit from corticosteroids to a greater extent compared to patients with the CAP2 subphenotype. We therefore conducted an exploratory analysis of heterogeneity of corticosteroid treatment effect in the COVID-19 cohort, with the caveat that corticosteroid exposure was not randomized. There was evidence of effect modification, such that corticosteroid exposure was associated with lower hazard of death in CAP1, but not in CAP2, unadjusted (P-value for interaction term = 0.002, Supplementary Table S15, Fig. 5) and adjusted for SOFA score, which was included to address indication bias (P-value for interaction term = 0.002, Supplementary Table S15). There was no interaction between corticosteroid exposure and SOFA score (as a continuous variable or when dichotomized about the median) for death within 90 days (P = 0.92), nor between corticosteroid exposure and shock (P = 0.35).

## Discussion

In this study, we identified two novel subphenotypes among patients hospitalized with CAP in northeastern Thailand by applying LCA to routinely collected clinical and laboratory variables. Survival differed between these subphenotypes after adjustment for measures of illness severity, and subphenotypes demonstrated distinct differences in plasma protein biomarkers of inflammation and metabolomic signatures. A PCM of four routinely available variables discriminated subphenotype assignment and was used to externally validate the subphenotypes in a multinational multicenter cohort in Southeast Asia. The subphenotypes were reproduced in a COVID-19 cohort in the U.S., overlapping moderately with previously identified COVID-19 subphenotypes, and, in an exploratory analysis of observational data, exhibited a differential response to corticosteroids. To our knowledge, this is the largest prospective study to identify and validate subphenotypes of CAP and the first outside of Europe. This report of subphenotypes is also the first in a resource-limited setting with diverse etiologies of respiratory infection and the first to compare metabolic profiles.

This study represents an important step toward understanding CAP heterogeneity globally, which is critical for development of context-specific precision treatment approaches. In both the derivation and external validation cohorts, CAP1 patients compared with CAP2 patients had higher modified SOFA scores, more abnormal vital signs, greater proportion with respiratory failure requiring mechanical ventilation, more frequent bacteremia, and higher mortality. The pathogen differences between subphenotypes were notable: Gram-negative causes of bacteremic pneumonia (*B. pseudomallei and K. pneumoniae*) predominated in CAP1 vs. a Gram-positive cause (*S. pneumoniae*) in CAP2. This suggests that pathogen type may contribute to CAP subphenotype assignment, possibly reflecting factors unique to this region with a high incidence of Gram-negative pneumonia.^24^^,^^33^^,^^41^^,^^42^

Though our dataset lacked the specific plasma proteins needed to directly apply subphenotyping schema published by other groups (e.g., IL-8, protein-C), the separation of clinical variables and plasma cytokines was similar to a pneumonia subphenotyping study in Europe,^11^ and to ARDS “hyperinflammatory” and “hypoinflammatory” subphenotypes.^5^^,^^28^^,^^43^ Further, a recent metagenomic analysis in a U.S.A. cohort found more frequent bacteremia in the “hyperinflammatory” sepsis subphenotype, which parallels our findings in CAP1.^6^ However, the specific pathogen breakdown between sepsis subphenotypes differed in their study compared with our CAP subphenotypes. Other notable difference from these prior studies is the lymphopenia present in CAP1, which has received significant attention as a potential marker of immune dysregulation in severe CAP.^44^ A unique feature of our study is the inclusion of lactate as a class-defining variable. Few prior subphenotyping studies were performed on cohorts with lactate available, and inclusion of lactate as a class-defining variable may alter subphenotype classification.^11^^,^^28^^,^^45^ Lactate measurements, e.g., using a point-of-care device, may be more readily available in LMICs than plasma protein biomarkers, making it a promising candidate as part of parsimonious classifier models in prospective studies. Future studies could measure lactate as well as variables included in published classifier models to more rigorously assess overlap of subphenotyping schema developed in different cohorts.^43^

Comparing metabolomic data between CAP subphenotypes is a novel aspect of our study, offering insights into host–pathogen interactions and systemic metabolic alterations during infection that may underpin the subphenotypes, which may represent novel opportunities for therapeutic intervention.^9^^,^^33^^,^^46^^,^^47^ We found that metabolic profiles differed between the subphenotypes in the Thai derivation cohort, both before and after adjustment for illness severity. CAP1 was characterized by increased glycolytic activity as evidenced by higher abundance of lactate, malate, pyruvate, and increased activity of the pentose phosphate pathway. A shift toward glycolysis has received significant attention in sepsis as a potential treatable trait and postulated to characterize a “hyperinflammatory” subphenotype of ARDS.^40^^,^^48^, ^49^, ^50^ Lipid dysregulation additionally distinguished the two subphenotypes. Metabolites decreased in CAP1 compared with CAP2 were almost exclusively from the Lipid metabolism super-pathway; lysophospholipids were the most depleted in CAP1. A recent study of metabolic subphenotypes of septic shock found low lysophospholipids and high plasma cytokines characterized the subgroup with highest mortality.^51^ A similar finding of low lysophospholipids was also found in the “hyperinflammatory” subphenotype of ARDS.^40^ Lysophospholipids perform immunomodulatory roles such as induction of adhesion molecule expression, release of chemotactic factors, and induction of monocyte chemotaxis, which could explain this finding in pneumonia at the intersection of acute lung injury and sepsis.^52^^,^^53^ Deeper characterization of dysregulated host metabolism in CAP subphenotypes is an important area of investigation and an exciting avenue to identify novel targets for host-directed therapies.^9^

In the COVID-19 cohort, the differential response to corticosteroids was intriguing, though exploratory because steroid treatment was not randomized, treatment paradigms differ between COVID-19 and other causes of CAP, and host responses between bacterial CAP and COVID-19 incompletely overlap.^54^, ^55^, ^56^, ^57^, ^58^, ^59^ We found that corticosteroid exposure was associated with lower mortality in CAP1 but not in CAP2. It is unlikely that this heterogeneity of treatment effect was due to differences in severity of illness alone, as neither SOFA score nor shock modified the association between corticosteroid exposure and 90-day mortality. This is the first study to demonstrate heterogeneity of treatment effect within a population hospitalized for infection whose subphenotypes were derived from an entirely different population, however, this aspect of our study is hypothesis-generating and should not be overinterpreted. Prospective evaluation of these subphenotypes in randomized controlled trials of CAP in diverse care settings and populations are required to confirm these findings and to guide context-specific approaches for adjunctive corticosteroid therapy in CAP.

Our study has several strengths. To the best of our knowledge, this is the largest prospective study to identify and validate subphenotypes of CAP and the first conducted in a tropical region with distinct host factors and etiologic pathogens. The Thai derivation cohort is uniquely large with 53 originating hospitals represented, and the SE Asian external validation cohort enrolled from 13 centers in three countries in SE Asia. Our dataset featured limited missing data, near-universal follow-up, and analyzed only variables from within the first 24 h after admission to the study hospital. Restricting class-defining variables to routinely collected clinical and laboratory data further adds potential usefulness to this subphenotyping approach, as they are likely to be available in resource-limited settings.

Our study does have important limitations. While our definition of CAP was pragmatic in a resource-limited setting, pneumonia misclassification is possible. Additionally, it is possible that illness severity was the “latent class” or underlying driver separating subphenotypes because the reported CAP subphenotypes were derived from clinical and laboratory variables that may represent final common pathways for many underlying biological processes, including those that impact illness severity. However, the prognostic significance of CAP1 independent of modified SOFA score argues against this, and the plasma metabolomic distinctions in the Thai derivation cohort were independent of modified SOFA score as well. Further, traditional illness severity scores (CURB-65 and modified SOFA) did not sufficiently discriminate CAP subphenotypes in the Thai derivation cohort. Infectious etiology may contribute to subphenotype assignment, though a pathogen was detected in a minority of patients in both SE Asia cohorts as is common in clinical practice. The metabolomic analyses were performed in a sample selected by pathogen for a separate study, which could introduce selection bias. However, the patients with and without metabolomic data were similar overall in the measured clinical variables and pathogen mix. Finally, the determination of subphenotype was cross-sectional; in the few studies examining subphenotype assignment over time, subphenotype assignment can be dynamic with potential prognostic and therapeutic implications.^60^ This is an important area of future research.

In summary, we have identified and externally validated two subphenotypes of patients with CAP in SE Asia with distinct inflammatory and metabolomic profiles and clinical outcomes. These subphenotypes were reproduced in a cohort of COVID-19 in the U.S. and were associated with differential responses to corticosteroids in a secondary analysis of observational data. These subphenotypes could form a foundation to understand CAP pathophysiology and develop future contextualized treatment approaches.

## Contributors

T.D.C., L.X., S.W.W., P.C., D.L., K.C.L., C.Y.L., N.V.V.C., M.J.C., M.R.O., P.K.B., L.R.Z., S.A.G., A.S., and T.E.W. participated in the study design and the conception of this work. V.H., G.W., D.L., P.P., D.F., K.C.L., C.Y.L., and N.V.V.C. contributed to the acquisition of the data, and V.H., D.L., L.X., S.A.G., and T.E.W. accessed and verified the data. T.D.C. led the data analysis and wrote the first draft of the manuscript. P.P., J.E.R., L.X., S.W.W., L.R.Z., D.F., D.L., S.A.G., A.S., and T.E.W. contributed to interpreting the data and editing the manuscript. All authors have read and approved the manuscript before submission.

## Data sharing statement

Deidentified demographic information and metabolomic data will be deposited in Metabolomics Workbench upon publication.

## Declaration of interests

We declare no competing interests.
