# Supplementary material for: Derivation and external validation of community-acquired pneumonia subphenotypes in Southeast Asia: a secondary analysis of prospective cohort studies
Source: eClinicalMedicine. 2025 Oct 24;90:103572. doi: 10.1016/j.eclinm.2025.103572 (PMC12595281; doi:10.1016/j.eclinm.2025.103572)
Supplement: Supplementary Figures and Tables [file mmc1.docx]

# **Supplementary material**

**Table of Contents**

| Supplementary Methods | Page 2 |
| --- | --- |
| Table S1. Class-defining variables | Page 6 |
| Table S2. Excluded variables | Page 7 |
| Table S3. Latent class analysis model fit | Page 8 |
| Table S4. Pathogens isolated in blood culture | Page 8 |
| Table S5. Log_2_ transformed plasma cytokine concentrations by subphenotype | Page 9 |
| Table S6. Linear regression for CAP1 vs CAP2 plasma cytokine concentrations | Page 9 |
| Table S7. Baseline variables among patients with metabolomic data available | Page 10 |
| Table S8. Pathogens isolated in blood culture among patients with metabolomic data available | Page 11 |
| Table S9. Top fifteen significant differentially abundant metabolites comparing CAP1 with CAP2 adjusted for age and sex | Page 12 |
| Table S10. Top fifteen significant differentially abundant metabolites comparing CAP1 with CAP2 adjusted for age, sex, and modified SOFA score | Page 13 |
| Table S11. Twenty-six significantly differentially enriched pathways comparing CAP1 with CAP2 adjusted for age, sex, and modified SOFA score | Page 14 |
| Table S12. Parsimonious classifier model performance for CAP1 vs CAP2 classification | Page 15 |
| Table S13. Overlap of original COVID-19 subgroups and CAP subphenotypes | Page 16 |
| Table S14. Baseline variables of adults with CAP due to COVID-19 receiving mechanical ventilation stratified by CAP subphenotype | Page 17 |
| Table S15. Comparison of death by 90 days in the COVID-19 cohort | Page 19 |
| Table S16. Hazard ratios for subphenotype and 90-day mortality in the COVID-19 cohort | Page 19 |
| Table S17. Plasma cytokine power calculations | Page 20 |
| Figure S1. Histogram of modified SOFA score by subphenotype | Page 21 |
| Figure S2. Metabolomic comparison CAP1 vs. CAP2 | Page 22 |
| Figure S3. Scatterplot of standardized mean differences and differences in proportions in derivation and external validation cohorts | Page 23 |
| Figure S4. Alluvial plot | Page 23 |
| Supplementary References | Page 24 |

**Supplementary methods**

# Subject selection in the derivation and external validation cohorts

The Thai derivation cohort drew from the parent Ubon-sepsis study, which prospectively enrolled patients ≥ 18 years old who were admitted to the general medical wards or the medical ICUs at Sunpasitthiprasong Hospital, Ubon Ratchathani, Thailand, from March 2013 to January 2017 with a primary diagnosis of infection made by the attending physician, who were within 24 hours of admission to the study hospital, and had at least three Surviving Sepsis Campaign criteria for sepsis documented in the medical record.^1^ Patients were excluded if diagnosed with hospital-acquired infections, had a previous hospitalization within the past 30 days, or were transferred from another hospital with a total duration of hospitalization > 72 hours. Among this cohort of patients with community-acquired infection, only those presenting with community-acquired pneumonia (CAP) defined by meeting all the following criteria were included: a) the presence of cough, sputum production, or shortness of breath, and b) primary admission diagnosis of pneumonia by the treating clinician after their interpretation of clinical and radiographic data, and c) final (discharge) diagnosis of pneumonia or melioidosis. Discharge diagnosis of melioidosis was included in the CAP criteria because it is common practice to record a melioidosis pneumonia patient as only “melioidosis” in the medical record. The SE Asian external validation cohort drew from the SEAICRN study, which employed similar methods to the Ubon-sepsis study at 13 sites in Thailand, Vietnam, and Indonesia from 2013 through 2015.^2^ CAP was defined in this cohort by meeting all of the following criteria: a) at least one respiratory symptom (dyspnea, cough, sputum production) for <14 days and b) pneumonia diagnosed by an attending physician.

*Procedures*

At enrollment into the Thai derivation cohort and the SE Asian external validation cohort, venous whole blood lactate was measured using a point-of-care device (Lactate Pro 2, Arkray, Australia). Data from the study hospital clinical laboratory used in this analysis included a white blood cell count (WBC) and differential, hemoglobin, platelet count, creatinine, and serum glucose obtained on the day of enrollment. Additional clinical and laboratory data were obtained from the patient's medical records. After enrollment, patients were treated according to local standard of care and followed until 28-days after enrollment.

*Clinical scores*

For the Thai derivation cohort and the SE Asian external validation cohort, a modified sequential organ failure assessment (modified SOFA) score was calculated for all subjects at the time of enrollment, given the absence of some data points such as inotrope and vasopressor agent doses and partial pressure of oxygen in arterial blood (PaO_2_). This modified SOFA score has been described previously.^1,3^ Separately, a previously published ordinal SpO_2_/FiO_2_ score of 0-4 was used as a class defining variable for latent class analysis in the derivation cohort: 0: >400, 1: >315 to <400, 2: >235 to <315 3:>150 to <235 4: <150.^4^ This scoring system does not require SpO2 <= 96% to ensure accuracy.^5^ CURB-65 (Confusion, Urea, Respiratory Rate, Blood Pressure, Age ≥ 65 years) has been validated for CAP mortality prediction in Southeast Asia.^6^

*Latent class analysis*

Baseline demographic data, clinical data (including comorbidities, initial vital signs, vasopressor requirements), and routine laboratory tests hypothesized to contribute to CAP host responses and/or reflect downstream effects of biological pathways activated in CAP were considered as class-defining variables in the LCA model; classification was conducted without consideration of clinical outcomes (Table S1).^7–11^ All clinical variables were assessed at screening within 24 hours of admission to the study hospital, and laboratory variables were assed day zero or day one of admission to the study hospital. Continuous variables with >10% missingness and categorical variables with <10% in a group were excluded.^12^ Variables were processed prior to LCA according to best practices.^13^ Right-skewed continuous variables were log_10_-transformed and left-skewed continuous variables were reflected then log_10_-transformed. Next, standardized z-scores were calculated for each continuous variable. A correlation matrix was used to assess correlation between variables based on Pearson's correlation coefficient and variables excluded if correlated >0·5 (Table S2). Pairwise correlation between variables was assessed, and the final 25 variables in Table S1 were used to perform latent class analysis. Sample sizes of at least 500 have been recommended in latent class analysis; for this study, sample size was adequate with 953 patients in the derivation cohort.^14^

Four latent class models were generated ranging from two to four subgroups. We then used the Vuong-Lo-Mendell-Rubin (VLMR) likelihood ratio test as our primary test for model fit based on prior reports.^15^ We also considered additional criteria, including the Bayesian Information Criteria (lower values suggest model parsimony), the entropy statistic (a measure of class separation, with optimal values greater than 0·8), class interpretability (the extent to which additional classes provided unique information), and class prevalence (preferring classes with at least 10% of the sample for improved replicability).^16^ A two-class model provided significantly better fit than a one-class model based on the VLMR test and model had higher entropy compared with a 3-class or 4-class model. The 3-class and 4-class models each had a subgroup containing less than the pre-specified 10% of the cohort. Therefore, a two-class model (CAP1 and CAP2) was chosen for further analysis (Table S3).

After a final LCA model was selected, patients were assigned to a respective subphenotype if their probability for class assignment was >0·5. Cox regression models were then developed to assess hazard of 28-day mortality by subphenotype both unadjusted and adjusted for age, sex, Charlson Comorbidity Index, modified SOFA score, and transfer status. Enrollment clinical data, including calculated scores, were summarized using proportions for discrete variables and medians and interquartile ranges (IQR) for continuous variables. Differences in variables between groups were evaluated by the chi-square test, Fisher exact test if counts in a cell were fewer than five, or Wilcoxon rank-sum test.

# Plasma protein biomarker assays

Prior to performing biomarker assays, power calculations were performed based on our preliminary data and prior studies of sepsis in Thailand.^17,18^ Using simulation-based power calculations, a sample size of 145 in each subphenotype was required to detect a 2-fold difference in biomarker concentrations between subphenotypes with 80% power (Table S17). After subphenotypes were identified via LCA, biomarker assays were performed on plasma samples obtained at the time of enrollment among a random subset of subjects selected from each subphenotype using a random number generator. The concentrations of IL-6, TNF-α, IL-1β, and IL-10 were measured by electrochemiluminescence multiplex assay (Meso Scale Discovery, Rockville, MD). Samples were diluted based on sensitivity of the assays and upper and lower limits of detection were determined by the manufacturer’s software for each plate. For sample concentrations below the lower limit of detection, a concentration was imputed between the lower limit of detection and 1/2 the lower limit of detection to maintain variability. Cytokines were log_2_ transformed for analysis. T-test of means was performed to compare log_2_ transformed cytokine concentrations between subgroups, and linear regression performed to compare log_2_ transformed cytokine concentrations between subgroups adjusted for modified SOFA score and transfer status (Tables S5 and S6, Figure 4A).

# Patient selection for metabolomic analyses

For a separate study,^19^ plasma from a random sampling of all patients with melioidosis (as determined by a positive culture for *Burkholderia pseudomallei* from any clinical sample) and plasma from random samplings of patients with *Escherichia coli*, *Staphylococcus aureus*, or *Klebsiella pneumoniae* bacteremia (the most common causes of community-acquired bacteremia) and a set of patients who had suspected infection but negative blood cultures and no positive culture for *B. pseudomallei* were selected for metabolomic analyses. Metabolomic analyses were performed on an additional random selection of CAP patients from each of the following infection etiology groups who were not sampled for the metabolomics of melioidosis study: *Streptococcus pneumoniae*, *E. coli*, *S. aureus,* and culture negative. Random samplings were performed within each infection etiology using the Stata function “runiform”. One hundred twenty-five subjects with metabolomic data available from the selection procedures above were present in the CAP cohort for the current study and comprised the sample for metabolomic analyses (Supplementary Table S7 and S8).

*Statistical analyses for metabolomics*

Metabolomics was performed by Metabolon, Inc., Morrisville, North Carolina on plasma samples from study participants.^19^ Sample preparation utilized the automated MicroLab STAR® system by the Hamilton Company, employing methanol precipitation to eliminate proteins and other macromolecules. Samples were analyzed using four distinct methods, including two separate reverse phase (RP)/UHPLC/MS/MS methods with positive ion mode electrospray ionization (ESI), one RP/UHPLC/MS/MS method with negative ion mode ESI, and one HILIC/UHPLC/MS/MS with negative ion mode ESI. Each method was optimized for specific metabolite retention and ionization properties through different injection solvents, chromatography, and mass spectrometer parameters. Experimental samples were randomized across the platform run, and quality control (QC) samples were evenly spaced between injections. Metabolon, Inc. conducted all QC procedures, and all samples passed QC before mass-spectrometry analysis. Metabolite identification was standardized through various curation processes. Pathway information was curated by Metabolon, Inc. Metabolites were measured in two batches each with unique patients from the Ubon-sepsis cohort with ten overlapping samples used to bridge between the two batches (“bridge subjects”). Metabolite data from these two batches were merged using bridge normalization, dividing each metabolite by the median value of the ten “bridge subjects.” Linear regression was performed with metabolite abundance as the dependent variable and subphenotype assignment as the independent variable, adjusted for age, sex, and modified SOFA score. For pathway analysis, the R package “globaltest” (version 5·50·0) was used comparing subphenotypes adjusted for the same covariables. For differential metabolite and pathway analyses, adjusted p-values were obtained using the Benjamini-Hochberg procedure for false discovery rate (FDR) control and significance was denoted at <0·05.

# Metabolomic analyses without modified SOFA

In the Thai derivation cohort, we compared the metabolomes of patients in CAP1 vs CAP2 adjusted for age and sex. Five hundred twenty-three metabolites were significantly differentially abundant comparing subgroups (388 increased in CAP1, 135 decreased in CAP1; Table S9; Supplementary Figure S2). Metabolites that were increased to the greatest degree in CAP1 compared with CAP2 were similar to the fully adjusted analysis, including those suggesting increased glycolytic activity (lactate, malate, pyruvate), metabolites from the Polyamine super-pathway (N1,N12-diacetylspermine, N('1)-acetylspermidine), and estrone-3-sulfate. Consistent with the fully adjusted analysis, metabolites that were decreased in CAP1 compared with CAP2 belonged predominantly to the Lipid super-pathway including 1-palmitoyl-GPE and 1-stearoyl-GPC. In pathway analysis using the global test, 62 of 67 pathways were significantly different comparing CAP1 with CAP2 adjusted for age and sex (Supplementary Figure S2).

# Development of a parsimonious classifier model

Classification of subphenotype assignment enables subphenotype validation in external cohorts and could have clinical utility for prognosis and predictive enrichment in clinical trials. Therefore, we sought to determine if a parsimonious set of variables could classify subphenotype assignment in the Thai derivation cohort. In 5-fold cross validation Least Absolute Shrinkage and Selection Operator (LASSO) regression using the full derivation CAP cohort, 15 of the original 25 class-defining variables were selected for inclusion in the model using the largest lambda for which mean squared error (MSE) is within one standard error of the minimal MSE. Because a parsimonious model with a smaller number of variables is preferred, the top variables selected by LASSO were considered: lactate, platelet count, vasopressor use [yes/no], heart rate, and bacteremia. These were also the variables with greatest standardized mean difference between the subphenotypes – a method of variable selection used by other groups.^10,20^ Next, logistic regression was applied in the Thai derivation cohort to develop the PCM using these variables selected by LASSO. The PCM was then tested for subphenotype identification via bootstrap internal validation with 200 replicates in the derivation cohort using the “validate” function in the R package “rms,” which gave an optimism-corrected C-statistic via Harrell’s bias correction.^21–23^ 95% confidence intervals were obtained via two-stage bootstrap sampling with 1,000 replicates in the second stage – 1,000 replicates were performed instead of 10,000 due to the high computation burden, which is an accepted practice in this setting.^24,25^ As bacteremia is unlikely to be useful to stratify patients early during a patient’s illness, bootstrap internal validation was performed with and without bacteremia in the model. The optimism-corrected C-statistic was similar with and without bacteremia: 0·98 (95% CI 0·97-0·99) with bacteremia and 0.97 (95% CI 0·96-0·98) without bacteremia. Therefore, the four-variable PCM (lactate, platelet count, vasopressor use, heart rate) was used: logit (p) = -11· 23413 + 2·312366 *vasopressors [yes/no] + 0· 09522902 *heart rate - 0·01817898*platelets (10^9^/L) + 0·5769045*lactate (mmol/L). The model was assessed for goodness of fit using Hosmer-Lemeshow chi-square analysis (p=0·96).^26^ A Youden index threshold representing optimal discrimination by maximizing sensitivity and specificity was then determined in the bootstrapped dataset (optimal cut-point = 0·2140947). Subsequently, the sensitivity and specificity of the parsimonious model to discriminate subphenotype assignment were calculated (Table S12). For performance in the bootstrap internal validation, median out-of-bag sensitivity and specificity across bootstrap replicates were reported.

*Sensitivity analysis for class imbalance*

To assess the potential impact of class imbalance on PCM performance, we conducted two sensitivity analyses by repeating the LASSO variable selection and logistic regression steps for PCM development after (1) downsampling the majority class (CAP2) and (2) upsampling the minority class (CAP1). For upsampling, CAP1 cases were sampled with replacement to match the number of CAP2 cases. For downsampling, CAP2 cases were randomly sampled without replacement to match the number of CAP1 cases. In both balanced datasets, we performed LASSO regression to identify the top variables retained in the model to classify CAP1 vs. CAP2. In the downsampled dataset, the five variables lactate, platelet count, vasopressor use, heart rate, and bacteremia were again the top variables retained in the model. In the upsampled dataset, respiratory rate was retained before vasopressor use, but otherwise the top variables were again selected. We then re-fit the logistic regression models using the four original predictor variables (venous lactate, platelet count, vasopressor use, heart rate) and evaluated model performance using bootstrap internal validation with 200 replicates. Model discrimination (optimism-corrected C-statistics) remained consistent across both sensitivity analyses and supported the robustness of the primary PCM: 0.968 in the upsampled dataset and 0.962 in the downsampled dataset.

# Comparison of four-variable PCM with illness severity measures

To assess if the four-variable PCM adds utility to traditional illness severity measures for discriminating subphenotype, the predictive ability of the four-variable PCM to discriminate subphenotype was compared to that of modified SOFA, lactate, and CURB-65 in the derivation cohort (Table S12). The procedures for PCM derivation, internal validation, and optimal cut-point identification were performed as described above, and model performance reported in Table S12. The optimism-corrected C-statistic for the four-variable PCM was higher than that of all three other scores (modified SOFA, lactate, and CURB-65) with no overlap of their 95% confidence intervals (Supplementary Table S12).

# **Supplementary Tables**

| **Table S1.** Class-defining variables for latent class analysis in the Thai derivation cohort (Ubon-sepsis) | |
| --- | --- |
| Variable |  |
| **Baseline demographics** | |
| Age- years | |
| Sex- male, female | |
| Body mass index- underweight, normal, overweight or obese | |
| **Comorbidities** | |
| Diabetes- yes, no | |
| Heart disease- yes, no | |
| Lung disease- yes, no | |
| Hypertension- yes, no | |
| **Symptoms** | |
| Symptom duration- days | |
| **Vital signs** | |
| Body temperature- °C | |
| Heart rate- beats per minute | |
| Respiratory rate- breaths per minute | |
| Systolic blood pressure- mmHg | |
| **Organ failure / life support** | |
| Mechanical ventilation- yes, no | |
| SpO_2_/FiO_2_ - ordinal scale 0-4 | |
| Vasopressors- yes, no | |
| **Routine laboratory biomarkers** | |
| Leukocyte count- cells x 10^-9^/L | |
| %neutrophils- % | |
| %monocytes- % | |
| %eosinophils- % | |
| Hemoglobin- g/dL | |
| Platelet count- platelets x 10^-9^/L | |
| Creatinine- mg/dL | |
| Lactate- mmol/L | |
| Blood glucose- mg/dL | |
| **Micro data** | |
| Bacteremia- yes, no | |
| Variables were processed prior to latent class analysis according to best practices as described in the supplementary methods. Continuous variables were log_10_-transformed and normalized, categorical variables with less than 10% in a category were collapsed. | |

**Table S2.** Additional variables considered for class definition with reason for exclusion

| Variable | Exclusion reason |
| --- | --- |
| **Comorbidities** |  |
| Liver disease | <10% in a group |
| Kidney disease | Correlated with Cr >0·5 |
| Cancer | <10% in a group |
| Dyslipidemia | <10% in a group |
| Stroke | <10% in a group |
| HIV | <10% in a group |
| Smoking | <10% in a group |
| Heavy alcohol use | <10% in a group |
| Chronic prednisone | <10% in a group |
| **Vital signs** |  |
| Jaundice | <10% in a group |
| Diastolic blood pressure | correlated with SBP >0·5 |
| Glasgow coma scale | Too little variation |
| **Routine laboratory biomarkers** |  |
| %lymphocytes | correlated with leukocyte count > 0·5 |
| %basophils | Too little variation |
| Hematocrit | Correlated with Hgb >0·5 |
| BUN | Correlated with Cr >0·5 |
| PaO2 | >10% missing |
| Albumin | >10% missing |
| Total bilirubin (or direct?) | >10% missing |
| Alkaline phosphatase | >10% missing |
| AST | >10% missing |
| ALT | >10% missing |
| PT | >10% missing |
| aPTT | >10% missing |
| INR | >10% missing |

**Table S3.** Model fit in the Thai derivation cohort using latent class analysis

| CAP cohort |  |  |  |  |  |  |  |
| --- | --- | --- | --- | --- | --- | --- | --- |
|  |  |  |  |  |  |  |  |
| Number of classes | BIC* | Entropy ^†^ | N1 | N2 | N3 | N4 | p-value ^‡^ |
| 2^§^ | 51604 | 0·87 | 141 | 812 |  |  | 0·03 |
| 3 | 50974 | 0·81 | 64 | 555 | 334 |  | 0·01 |
| 4 | 50727 | 0·78 | 332 | 195 | 348 | 78 | 0·35 |

*Bayesian information criterion (BIC) is a criterion for model selection with lower values suggesting model parsimony. BIC is sample size-adjusted.

^†^ Entropy is an index of how well the classes are separated. It ranges from zero to one and values of 0·8 and up are generally considered a sign of a useful model.

^‡^ Vuong-Lo-Mendell-Rubin p-value tests whether the number of classes provides improved model fit compared to a model using one fewer class

^§^ A two-class model provided significantly better fit than a one-class model based on the VLMR test and had higher entropy compared with a 3-class or 4-class model. The 3-class and 4-class models each had a subgroup containing less than the pre-specified 10% of the cohort. Therefore, a two-class model (CAP1 and CAP2) was chosen for further analysis.

**Table S4**: Pathogens grown in blood culture by subphenotype among bacteremic patients

|  | Thai derivation cohort  (Ubon-sepsis) | |  | SE Asian external validation cohort  (SEAICRN) | |
| --- | --- | --- | --- | --- | --- |
| Pathogen count (%) | CAP1  (n = 56) | CAP2 (n=61) |  | CAP1  (n=12) | CAP2  (n=16) |
| Acinetobacter spp. | 3 (5·1) | 1 (1·6) |  | 2 (16·6) | 2 (12·5) |
| Aeromonas spp. | 1 (1·7) | 0 (0) |  | 0 (0) | 0 (0) |
| Burkholderia pseudomallei | 24 (40·7) | 20 (31·3) |  | 2 (16·6) | 1 (6·3) |
| Bacillus spp. | 0 (0) | 0 (0) |  | 0 (0) | 1 (6·3) |
| Coagulase-negative staphylococci | 0 (0) | 0 (0) |  | 4 (33·3) | 1 (6·3) |
| Coagulase-positive staphylococci | 3 (5·1) | 5 (7·8) |  | 0 (0) | 1 (6·3) |
| Corynebacterium spp. | 0 (0) | 0 (0) |  | 0 (0) | 1 (6·3) |
| Cryptococcus neoformans | 0 (0) | 1 (1·6) |  | 0 (0) | 0 (0) |
| Diphtheroid spp. | 0 (0) | 0 (0) |  | 0 (0) | 1 (6·3) |
| E. coli | 8 (13·6) | 9 (14·1) |  | 1 (8·3) | 0 (0) |
| Enterobacter spp. | 0 (0) | 0 (0) |  | 0 (0) | 2 (12·5) |
| Klebsiella pneumoniae | 9 (15·3) | 1 (1·6) |  | 1 (8·3) | 0 (0) |
| Nocardia spp. | 0 (0) | 1 (1·6) |  | 0 (0) | 0 (0) |
| Penicillium marneffei | 1 (1·7) | 0 (0) |  | 0 (0) | 0 (0) |
| Proteus mirabilis | 1 (1·7) | 0 (0) |  | 0 (0) | 0 (0) |
| Pseudomonas spp. | 1 (1·7) | 5 (7·8) |  | 0 (0) | 0 (0) |
| Salmonella spp. | 0 (0) | 3 (4·7) |  | 0 (0) | 1 (6·3) |
| Streptococcus pneumoniae | 4 (6·8) | 11 (17·2) |  | 1 (8·3) | 2 (12·5) |
| Other Streptococcus spp. | 4 (6·8) | 7 (10·9) |  | 1 (8·3) | 2 (12·5) |
| Other Gram-negative | 0 (0) | 0 (0) |  | 0 (0) | 1 (6·3) |

| **Table S5**: Log_2_ transformed plasma cytokine concentrations (log_2_ pg/ml) in the Thai derivation cohort by subphenotype | | | |
| --- | --- | --- | --- |
| Cytokine, mean (95% CI) | CAP1 (n = 141) | CAP2 (n=141) | P-value* |
| IL-10 | 5·8 (5·28-6·32) | 1·94 (1·64-2·24) | <0·001 |
| IL-1β | 1·72 (1·28-2·16) | -0·47 (-0·65--0·28) | <0·001 |
| IL-6 | 10·2 (9·62-10·79) | 5·21 (4·84-5·58) | <0·001 |
| TNF-α | 4·8 (4·45-5·14) | 2·69 (2·47-2·92) | <0·001 |
| *Abbreviations*: CI = confidence interval *P-value obtained via unadjusted linear regression. | | | |

| **Table S6.** Linear regression coefficients for CAP1 vs CAP2 with  log_2_ cytokine as the dependent variable in the Thai derivation cohort | | | | |
| --- | --- | --- | --- | --- |
|  | **Unadjusted** | | **Adjusted*** | |
| **Biomarker** | **Coefficient  (95% CI)** | **P-value** | **Coefficient  (95% CI)** | **P-value** |
| IL-10 | 3·86 (3·26-4·45) | <0·001 | 2·08 (1·32-2·83) | <0·001 |
| IL-1β | 2·19 (1·71-2·66) | <0·001 | 1·71 (1·08-2·35) | <0·001 |
| IL-6 | 5·0 (4·31-5·69) | <0·001 | 3·77 (2·74-4·81) | <0·001 |
| TNF-α | 2·10 (1·69-2·51) | <0·001 | 0·97 (0·45-1·5) | <0·001 |
| Abbreviations: CI = confidence interval  *Adjusted for modified SOFA score and transfer status | | | | |

| **Table S7**: Baseline variables stratified by subphenotype among patients in the Thai derivation cohort with metabolomic data available | | | |
| --- | --- | --- | --- |
| Variable | CAP1 (n = 50) | CAP2 (n=75) | P-value |
| **Baseline demographics** |  |  |  |
| Age in years | 58 (45-69) | 64 (46-76) | 0·12 |
| Female sex | 21 (42·0) | 31 (41·3) | 0·941 |
| Body mass index, kg/m^2^ |  |  | 0·66 |
| <18·5 | 12 (24·0) | 15 (20·0) |  |
| 18·5 - 24·9 | 32 (64·0) | 48 (64·0) |  |
| 25 - 29·9 | 5 (10·0) | 7 (9·3) |  |
| > 30 | 1 (2·0) | 5 (6·7) |  |
| **Comorbidities** |  |  |  |
| Diabetes | 21 (42·0) | 26 (34·7) | 0·407 |
| Heart disease | 5 (10·0) | 3 (4·0) | 0·179 |
| Lung disease | 2 (4·0) | 15 (20·0) | 0·011 |
| Smoking | 2 (4·0) | 1 (1·3) | 0·34 |
| Hypertension | 8 (16·0) | 23 (30·7) | 0·063 |
| Liver disease | 4 (8·0) | 0 (0·0) | 0·013 |
| Kidney disease | 2 (4·0) | 9 (12·0) | 0·122 |
| Cancer | 2 (4·0) | 1 (1·3) | 0·34 |
| HIV | 0 (0) | 0 (0) | 1·0 |
| **Symptoms** |  |  |  |
| Symptom duration, days |  |  | 0·488 |
| <=2 days | 18 (36·0) | 26 (34·7) |  |
| 3-7 days | 26 (52·0) | 34 (45·3) |  |
| >7 days | 6 (12·0) | 15 (20·0) |  |
| **Vital signs** |  |  |  |
| Body temperature, °C | 38 (37-39) | 37 (37-39) | 0·88 |
| Heart rate beats per minute | 113 (100-128) | 96 (88-112) | <0·001 |
| Respiratory rate, breaths per minute | 24 (22-29) | 20 (20-24) | <0·001 |
| Systolic blood pressure, mmHg | 98 (91-120) | 114 (104-140) | <0·001 |
| **Organ failure / life support** |  |  |  |
| Mechanical ventilation | 45 (90·0) | 31 (41·3) | <0·001 |
| Modified SOFA | 9 (6-12) | 3 (1-5) | <0·001 |
| SpO_2_ / FiO_2_ ordinal score | 3 (2-4) | 2 (0-3) | <0·001 |
| Vasopressors | 26 (52·0) | 12 (16·0) | <0·001 |
| **Routine laboratory biomarkers** |  |  |  |
| Leukocyte count, x 10^-9^ /L | 9090 (5100-18360) | 14100 (11000-17680) | 0·010 |
| %neutrophils | 87 (77-93) | 87 (76-91) | 0·87 |
| %monocytes | 3 (1-4) | 4 (2-8) | 0·008 |
| %eosinophils | 0 (0-0) | 0 (0-1) | 0·32 |
| %lymphocytes | 8 (4-13) | 8 (5-12) | 0·97 |
| %basophils | 0 (0-0) | 0 (0-0) | 0·72 |
| Hemoglobin, g/dL | 10 (10-12) | 10 (9-12) | 0·92 |
| Platelet count, x 10^-9^ /L | 141000 (76000-187000) | 249000 (159000-325000) | <0·001 |
| Creatinine, mg/dL | 2 (1-3) | 1 (1-2) | 0·001 |
| Lactate, mmol/L | 5 (3-11) | 2 (1-2) | <0·001 |
| Blood glucose, mg/dL | 182 (128-249) | 160 (119-225) | 0·30 |
| **Micro data** |  |  |  |
| Bacteremia | 43 (86·0) | 42 (56·0) | <0·001 |
| **Transferred from outside facility** | 46 (92·0) | 60 (80·0) | 0·067 |
| Continuous variables are presented as median (interquartile range) and categorical data are presented as n (%). P-values obtained using Wilcoxon rank-sum test for continuous variables and chi-square or Fisher exact test for categorical variables. | | | |

**Table S8.** Pathogens grown in blood culture among patients in the Thai derivation cohort with metabolomic data available

| **Infection** | **Total** | **CAP1** | **CAP2** |
| --- | --- | --- | --- |
|  | **(N=125)** | **(N=50)** | **(N=75)** |
| Negative blood culture | 40 (32·0) | 7 (14·0) | 33 (44·0) |
| *E. coli* | 14 (11·2) | 6 (12·0) | 8 (10·7) |
| *K. pneumoniae* | 7 (5·6) | 7 (14·0) | 0 (0·0) |
| *B. pseudomallei* | 44 (35·2) | 24 (48·0) | 20 (26·7) |
| *Pneumococcus* | 12 (9·6) | 3 (6·0) | 9 (12·0) |
| *S. aureus* | 8 (6·4) | 3 (6·0) | 5 (6·7) |

**Table S9.** Top fifteen significant differentially abundant metabolites comparing CAP1 with CAP2 in the Thai derivation cohort adjusted for age and sex (508 additional significant differentially abundant metabolites are not shown here).

| Biochemical | P-value | Adjusted*  p-value | Super-pathway | Pathway |
| --- | --- | --- | --- | --- |
| **Increased in CAP1 vs CAP2** |  |  |  |  |
| estrone 3-sulfate | 2·18 ×10^-15^ | 2·13 ×10^-13^ | Lipid | Estrogenic Steroids |
| sedoheptulose | 3·07 ×10^-14^ | 2·40 ×10^-12^ | Carbohydrate | Carbohydrate |
| malate | 4·06 ×10^-13^ | 2·27 ×10^-11^ | Energy | Energy |
| lactate | 8·36 ×10^-13^ | 3·13 ×10^-11^ | Carbohydrate | Carbohydrate |
| pyruvate | 2·01 ×10^-11^ | 4·36 ×10^-10^ | Carbohydrate | Carbohydrate |
| androstenediol (3alpha, 17alpha)   monosulfate | 3·60 ×10^-10^ | 4·61 ×10^-9^ | Lipid | Androgenic Steroids |
|  |  |  |  |  |
| **Decreased in CAP1 vs CAP2** |  |  |  |  |
| 1-palmitoyl-GPC (16:0) | 7·63 ×10^-20^ | 5·96 ×10^-17^ | Lipid | Lysophospholipid and Lysoplasmalogen |
| 1-stearoyl-GPC (18:0) | 2·12 ×10^-19^ | 8·27 ×10^-17^ | Lipid | Lysophospholipid and Lysoplasmalogen |
| 1-(1-enyl-palmitoyl)-GPC (P-16:0) | 2·52 ×10^-18^ | 6·58 ×10^-16^ | Lipid | Lysophospholipid and Lysoplasmalogen |
| 6-bromotryptophan | 1·84 ×10^-16^ | 3·60 ×10^-14^ | Amino Acid | Tryptophan Metabolism |
| 1-oleoyl-GPC (18:1) | 7·84 ×10^-16^ | 1·02 ×10^-13^ | Lipid | Lysophospholipid and Lysoplasmalogen |
| 1-palmitoyl-GPE (16:0) | 1·35 ×10^-13^ | 8·12 ×10^-12^ | Lipid | Lysophospholipid and Lysoplasmalogen |
| 1-palmitoyl-GPG (16:0) | 4·65 ×10^-13^ | 2·27 ×10^-11^ | Lipid | Lysophospholipid and Lysoplasmalogen |
| sphingomyelin (d18:1/20:0,   d16:1/22:0) | 2·28 ×10^-11^ | 4·68 ×10^-10^ | Lipid | Sphingomyelins and Ceramide PEs |
| N-oleoyltaurine | 3·21 ×10^-9^ | 3·35 ×10^-8^ | Lipid | Endocannabinoid |
| *P-value adjusted using Benjamini-Hochberg adjustment to control false discovery rate | | | | |

**Table S10.** Top fifteen significant differentially abundant metabolites comparing CAP1 with CAP2 in the Thai derivation cohort adjusted for age, sex, and modified SOFA score (108 additional significant differentially abundant metabolites are not shown here).

| Biochemical | P-value | Adjusted*  p-value | Super-pathway | Pathway |
| --- | --- | --- | --- | --- |
| **Increased in CAP1 vs CAP2** |  |  |  |  |
| androstenediol (3alpha, 17alpha)   monosulfate (2) | 5·72 ×10^-8^ | 1·57 ×10^-5^ | Lipid | Androgenic Steroids |
| lactate | 2·86 ×10^-7^ | 3·19 ×10^-5^ | Carbohydrate | Carbohydrate |
| sedoheptulose | 4·01 ×10^-7^ | 3·92 ×10^-5^ | Carbohydrate | Carbohydrate |
| pyruvate | 1·99 ×10^-6^ | 1·41 ×10^-4^ | Carbohydrate | Carbohydrate |
| malate | 4·03 ×10^-6^ | 2·63 ×10^-4^ | Energy | Energy |
| estrone 3-sulfate | 5·92 ×10^-6^ | 3·39 ×10^-4^ | Lipid | Estrogenic Steroids |
| **Decreased in CAP1 vs CAP2** |  |  |  |  |
| 1-palmitoyl-GPC (16:0) | 5·26 ×10^-8^ | 1·57 ×10^-5^ | Lipid | Lysophospholipid and Lysoplasmalogen |
| 1-stearoyl-GPC (18:0) | 7·38 ×10^-8^ | 1·57 ×10^-5^ | Lipid | Lysophospholipid and Lysoplasmalogen |
| 1-palmitoyl-GPE (16:0) | 8·04 ×10^-8^ | 1·57 ×10^-5^ | Lipid | Lysophospholipid and Lysoplasmalogen |
| 6-bromotryptophan | 1·36 ×10^-7^ | 2·12 ×10^-5^ | Amino Acid | Tryptophan Metabolism |
| 1-palmitoyl-GPG (16:0) | 1·75 ×10^-7^ | 2·28 ×10^-5^ | Lipid | Lysophospholipid and Lysoplasmalogen |
| 1-oleoyl-GPC (18:1) | 8·30 ×10^-7^ | 7·22 ×10^-5^ | Lipid | Lysophospholipid and Lysoplasmalogen |
| 1-(1-enyl-palmitoyl)-GPC (P-16:0) | 1·87 ×10^-6^ | 1·41 ×10^-4^ | Lipid | Lysophospholipid and Lysoplasmalogen |
| sphingomyelin (d18:1/20:0, d16:1/  22:0) | 6·07 ×10^-6^ | 3·39 ×10^-4^ | Lipid | Sphingomyelins and Ceramide PEs |
| N-oleoyltaurine | 7·59 ×10^-6^ | 3·96 ×10^-4^ | Lipid | Endocannabinoid |
| *P-value adjusted using Benjamini-Hochberg adjustment to control false discovery rate | | | | |

**Table S11.** Twenty-six significantly differentially enriched pathways comparing CAP1 with CAP2 in the Thai derivation cohort adjusted for age, sex, and modified SOFA score. Benjamini-Hochberg adjusted p-values, super-pathways, and total numbers of metabolites in each pathway are displayed.

| Pathway | Super-pathway | Adjusted  p-value* | Total # of  metabolites |
| --- | --- | --- | --- |
| Lysophospholipid and Lysoplasmalogen | Lipid | 1·36E-04 | 19 |
| Fatty Acid Metabolism (Acyl Choline) | Lipid | 6·66E-04 | 3 |
| Endocannabinoid | Lipid | 0·004 | 6 |
| Sphingolipid Synthesis and Sphingosines | Lipid | 0·004 | 5 |
| Phospholipid, Inositol and Glycerolipid   Metabolism, and Phosphatidylserine (PS) | Lipid | 0·004 | 12 |
| Energy | Energy | 0·005 | 10 |
| Polyamine Metabolism | Amino Acid | 0·005 | 9 |
| Fatty Acid Metabolism (Acyl Carnitine,   Monounsaturated) | Lipid | 0·009 | 10 |
| Leucine, Isoleucine and Valine Metabolism | Amino Acid | 0·009 | 27 |
| Medium Chain Fatty Acid | Lipid | 0·009 | 10 |
| Carbohydrate | Carbohydrate | 0·011 | 26 |
| Corticosteroids | Lipid | 0·011 | 5 |
| Phosphatidylcholine (PC) | Lipid | 0·011 | 16 |
| Pregnenolone Steroids | Lipid | 0·011 | 7 |
| Sphingomyelins and Ceramide PEs | Lipid | 0·011 | 30 |
| Tyrosine Metabolism | Amino Acid | 0·011 | 17 |
| Lysine Metabolism | Amino Acid | 0·012 | 20 |
| Androgenic Steroids | Lipid | 0·022 | 16 |
| Secondary Bile Acid Metabolism | Lipid | 0·034 | 18 |
| Alanine and Aspartate Metabolism | Amino Acid | 0·036 | 7 |
| Lactosylceramides (LCER) | Lipid | 0·036 | 4 |
| Tryptophan Metabolism | Amino Acid | 0·036 | 20 |
| Hemoglobin and Porphyrin Metabolism | Cofactors and Vitamins | 0·043 | 6 |
| Fatty Acid Metabolism (Acyl Carnitine,   Long Chain Saturated) | Lipid | 0·045 | 8 |
| Glutathione Metabolism | Amino Acid | 0·048 | 7 |
| Fatty Acid Metabolism (Acyl Carnitine,   Polyunsaturated) | Lipid | 0·050 | 6 |
| *P-value adjusted using Benjamini-Hochberg adjustment to control false discovery rate | | | |

| **Table S12.** Parsimonious classifier model performance for CAP1 vs CAP2 classification in the Thai derivation cohort. | | |
| --- | --- | --- |
|  | **Derivation** | **Bootstrap internal validation*** |
|  | **(N=953)** | **(N=953)** |
| **Four-variable PCM: Lactate, platelet count, vasopressor use [yes/no], heart rate^†^** |  |  |
| C-statistic (95% CI) | 0·97 (0·96-0·98) | 0·97 (0·96 - 0·98) |
| Sensitivity^‡^ | 0·94 | 0·89 |
| Specificity | 0·89 | 0·92 |
| **Modified SOFA score** |  |  |
| C-statistic (95% CI) | 0·89 (0·87-0·92) | 0·89 (0·87 - 0·92) |
| Sensitivity | 0·82 | 0·72 |
| Specificity | 0·78 | 0·87 |
| **CURB-65** |  |  |
| C-statistic (95% CI) | 0·68 (0·63-0·73) | 0·68 (0·62 - 0·73) |
| Sensitivity | 0·44 | 0·43 |
| Specificity | 0·81 | 0·81 |
| **Venous lactate** |  |  |
| C-statistic (95% CI) | 0·89 (0·86-0·92) | 0·89 (0·86 - 0·92) |
| Sensitivity | 0·91 | 0·79 |
| Specificity | 0·70 | 0·79 |
| *Bootstrap internal validation was performed with 200 replicates. The optimism-corrected C-statistic via Harrell’s bias correction and median out-of-bag sensitivity and specificity are shown.  ^†^ Model equation: logit (p) = -11· 23413 + 2·312366 *vasopressors [yes/no] + 0· 09522902 *heart rate - 0·01817898*platelets (10^9^/L) + 0·5769045*lactate (mmol/L).  ^‡^ Sensitivity and specificity were calculated at the optimal cut-point that maximized the Youden index. | | |

| **Table S13.** Overlap of original COVID-19 classes and CAP subphenotypes by applying four-variable PCM | | | |
| --- | --- | --- | --- |
| **COVID-19 classes** | **CAP subphenotypes*** | |  |
|  | **CAP1** | **CAP2** | **Total** |
| **CARDS class 2** | 109 | 32 | 141 |
| **CARDS class 1** | 99 | 232 | 331 |
| **Total** | 208 | 264 | 472 |
| *Abbreviations:* CAP = community-acquired pneumonia, PCM = parsimonious classifier model. CARDS = Covid-19 – related ARDS.  *CAP subphenotypes were identified by applying the four-variable parsimonious classifier model (lactate, platelet count, vasopressor use [yes/no], heart rate) to the COVID-19 cohort (CAP1 and CAP2). CARDS class 1 and 2 were the subphenotypes identified in the original COVID-19 subphenotyping study.^27^ Cohen's Kappa for agreement is 0·42. | | | |

| **Table S14.** Baseline variables of adults with COVID-19 receiving mechanical ventilation stratified by CAP subphenotype | | | | |
| --- | --- | --- | --- | --- |
| Variable | CAP1 (n=208) | CAP2 (n=264) | Total (n=472) | P-value |
| **Demographics** |  |  |  |  |
| Age, years | 67 (54-75) | 64 (55-72) | 65 (55-73) | 0·17 |
| Sex, male | 142 (68·3) | 173 (65·5) | 315 (66·7) | 0·53 |
| Race |  |  |  |  |
| White | 45 (25·7) | 60 (24·6) | 105 (25·1) | 0·79 |
| Black | 39 (22·3) | 54 (22·1) | 93 (22·2) | 0·97 |
| Hispanic | 111 (58·7) | 165 (65·0) | 276 (62·3) | 0·18 |
| Body mass index | 28 (25-32) | 30 (26-34) | 29 (26-33) | 0·023 |
| **Comorbidities** |  |  |  |  |
| Hypertension | 145 (69·7) | 177 (67·0) | 322 (68·2) | 0·54 |
| Diabetes | 93 (44·7) | 112 (42·4) | 205 (43·4) | 0·62 |
| Heart disease | 49 (23·6) | 67 (25·4) | 116 (24·6) | 0·65 |
| Smoking | 44 (23·4) | 51 (20·7) | 95 (21·9) | 0·51 |
| **Symptoms** |  |  |  |  |
| Symptom duration, days | 7 (3-9) | 7 (4-8) | 7 (4-9) | 0·54 |
| Time from admission to intubation, days | 8 (4-12) | 8 (5-13) | 8 (4-13) | 0·18 |
| **Vital signs** |  |  |  |  |
| Body temperature, °C | 38 (37-39) | 38 (37-38) | 38 (37-39) | 0·21 |
| Heart rate, beats per minute | 112 (111-136) | 101 (88-114) | 110 (95-125) | <0·001 |
| Respiratory rate, breaths per minute | 33 (28-40) | 32 (26-38) | 32 (26-38) | 0·028 |
| Systolic blood pressure, mmHg | 86 (75-94) | 89 (80-97) | 88 (78-95) | <0·001 |
| **Laboratory data** |  |  |  |  |
| Leukocyte count, x 10^-9^ /L | 13 (8-19) | 12 (9-15) | 12 (9-17) | 0·26 |
| Lymphopenia* | 128 (61·5) | 159 (60·2) | 287 (60·8) | 0·084 |
| Platelet count, x 10^-9^ /L | 196 (140-249) | 273 (198-346) | 234 (168-304) | <0·001 |
| Creatinine, mg/dL | 2 (1-3) | 1 (1-2) | 2 (1-3) | 0·002 |
| Bicarbonate, mmol/L | 20 (16-23) | 21 (19-24) | 21 (17-23) | <0·001 |
| Glucose, mg/dL | 168 (123-239) | 154 (110-210) | 158 (116-225) | 0·026 |
| Sodium, mmol/L | 139 (134-143) | 138 (135-142) | 138 (135-143) | 0·87 |
| Total bilirubin, mg/dL | 1 (0-1) | 1 (0-1) | 1 (0-1) | 0·006 |
| Albumin, g/dL | 3 (3-4) | 3 (3-3) | 3 (3-4) | 0·53 |
| Lactate, mmol/L | 3 (2-5) | 2 (1-2) | 2 (1-3) | <0·001 |
| Troponin, ng/L | 54 (22-150) | 28 (13-80) | 38 (16-98) | <0·001 |
| IL-6, pg/ml | 185 (42-315) | 105 (30-218) | 126 (33-315) | <0·001 |
| Procalcitonin, ng/ml | 1 (0-4) | 1 (0-2) | 1 (0-2) | <0·001 |
| Ferritin, ng/ml | 1232 (738-2427) | 1142 (625-2275) | 1179 (664-2327) | 0·13 |
| Lactate dehydrogenase, U/L | 694 (522-977) | 586 (446-731) | 620 (472-850) | <0·001 |
| D-dimer, mcg/ml | 6 (2-20) | 4 (2-16) | 4 (2-20) | 0·020 |
| **Ventilator data** |  |  |  |  |
| PaO2/FiO2 | 130 (90-196) | 142 (91-197) | 138 (90-197) | 0·55 |
| Vt/PBW, ml/kg | 6 (6-7) | 6 (6-7) | 6 (6-7) | 0·33 |
| PEEP, cm H2O | 12 (10-16) | 12 (10-15) | 12 (10-15) | 0·85 |
| Compliance, ml/cm H2O | 26 (22-33) | 27 (22-33) | 27 (22-33) | 0·57 |
| Ventilatory ratio | 2 (2-3) | 2 (1-2) | 2 (1-3) | 0·045 |
| **Therapies** |  |  |  |  |
| Any corticosteroid | 148 (71·2) | 170 (64·4) | 318 (67·4) | 0·12 |
| Vasopressors | 200 (96·2) | 199 (75·4) | 399 (84·5) | <0·001 |
| Renal replacement therapy | 68 (32·9) | 79 (30·3) | 147 (31·4) | 0·55 |
| Neuromuscular blockade | 81 (38·9) | 75 (28·5) | 156 (33·1) | 0·017 |
| Prone positioning | 41 (19·7) | 68 (25·9) | 109 (23·1) | 0·12 |
| ECMO | 7 (3·4) | 3 (1·1) | 10 (2·1) | 0·096 |
| **SOFA score** | 8 (7-10) | 7 (5-8) | 7 (6-9) | <0·001 |
| **Mortality** | 126 (60·9) | 126 (47·9) | 252 (53·6) | 0·005 |
| *Abbreviations*: CAP = community-acquired pneumonia; ECMO = extracorporeal membrane oxygenation; PBW = predicted body weight; PEEP = positive end-expiratory pressure; SOFA = Sequential Organ Failure Assessment. Continuous variables are presented as median (interquartile range) and categorical data are presented as n (%). P-values obtained using Wilcoxon rank-sum test for continuous variables and chi-square or Fisher exact test for categorical variables.  *Lymphopenia was defined as absolute lymphocyte count <1,000 cells/µL | | | | |

| **Table S15.** Comparison of death by 90 days in subgroups stratified by corticosteroid exposure in CAP subphenotypes applied to the COVID-19 NYC cohort | | | |
| --- | --- | --- | --- |
| **Corticosteroid use** | **Subgroup** | |  |
|  | **CAP1** | **CAP2** | **P-value*** |
| **Yes** | 81/148 (55%) | 88/170 (52%) | 0·002 |
| **No** | 45/60 (75%) | 37/94 (39%) |  |
| *Abbreviations:* CAP = community-acquired pneumonia.  *P-value represents the interaction between CAP subphenotype and steroid exposure in Cox regression with death within 90 days as the dependent variable. | | | |

| **Table S16.** Hazard ratios for 90-day mortality comparing CAP1 with CAP2 in the COVID-19 cohort | | | | |
| --- | --- | --- | --- | --- |
|  | **Unadjusted** | | **Adjusted** | |
| **Outcome** | **HR (95% CI)** | **P-value** | **HR (95% CI)** | **P-value** |
| **90-day mortality** | 1·39 (1·08-1·78) | 0·011 | 1·22 (0·92-1·61) | 0·17 |
| *Hazard ratios were calculated with the use of Cox regression, unadjusted and adjusted for age, sex, SOFA score, and symptom duration. | | | | |

| **Table S17.** A priori sample size estimation to achieve 80% power to detect plasma cytokine concentration differences between subphenotypes based on cytokines measured prior to the present study. | |
| --- | --- |
| Effect size | Sample size per group |
| **Power calculation using SD of IL8 in Ubon-sepsis CAP patients by subphenotype (SD* for CAP1 = 3·25; SD for CAP2 = 2·77)** | |
|  |  |
| 1·2-fold | 2071 |
| 1·5-fold | 420 |
| 2-fold | 145 |
| 4-fold | 37 |
|  |  |
| **Power calculation using SD of IL8 in full Ubon-sepsis cohort (SD = 3·07)** | |
| 1·2-fold | 2140 |
| 1·5-fold | 434 |
| 2-fold | 149 |
| 4-fold | 38 |
|  |  |
| **Power calculation using SD of IL6 in melioidosis pneumonia cohort (SD = 3·07)** | |
| 1·2-fold | 1012 |
| 1·5-fold | 206 |
| 2-fold | 71 |
| 4-fold | 19 |
| *Abbreviations*: CAP = community-acquired pneumonia. SD = standard deviation.  *Standard deviation is on log_2_ scale, and plasma cytokines input into power calculation log_2_-transformed. | |

# **Supplementary Figures**

**Figure S1.** Histogram of modified SOFA score for CAP1 and CAP2 in the Thai derivation cohort.

**Figure S2.** (*a*) Heatmap of 523 significantly differentially abundant metabolites between patients in the Thai derivation cohort comparing CAP1 with CAP2, adjusted for age and sex. (*b*) Volcano plot comparing CAP1 with CAP2 adjusted for age and sex. Significant metabolites after Benjamini-Hochberg adjustment are highlighted; red: increased abundance, blue: decreased abundance. (*c*) Radial barplot of 62 significant pathways, obtained from comparing CAP1 with CAP2 adjusting for age and sex. Height of the bars represents negative log_10_ of Benjamini-Hochberg procedure adjusted p-values.

**a**

**b**

**
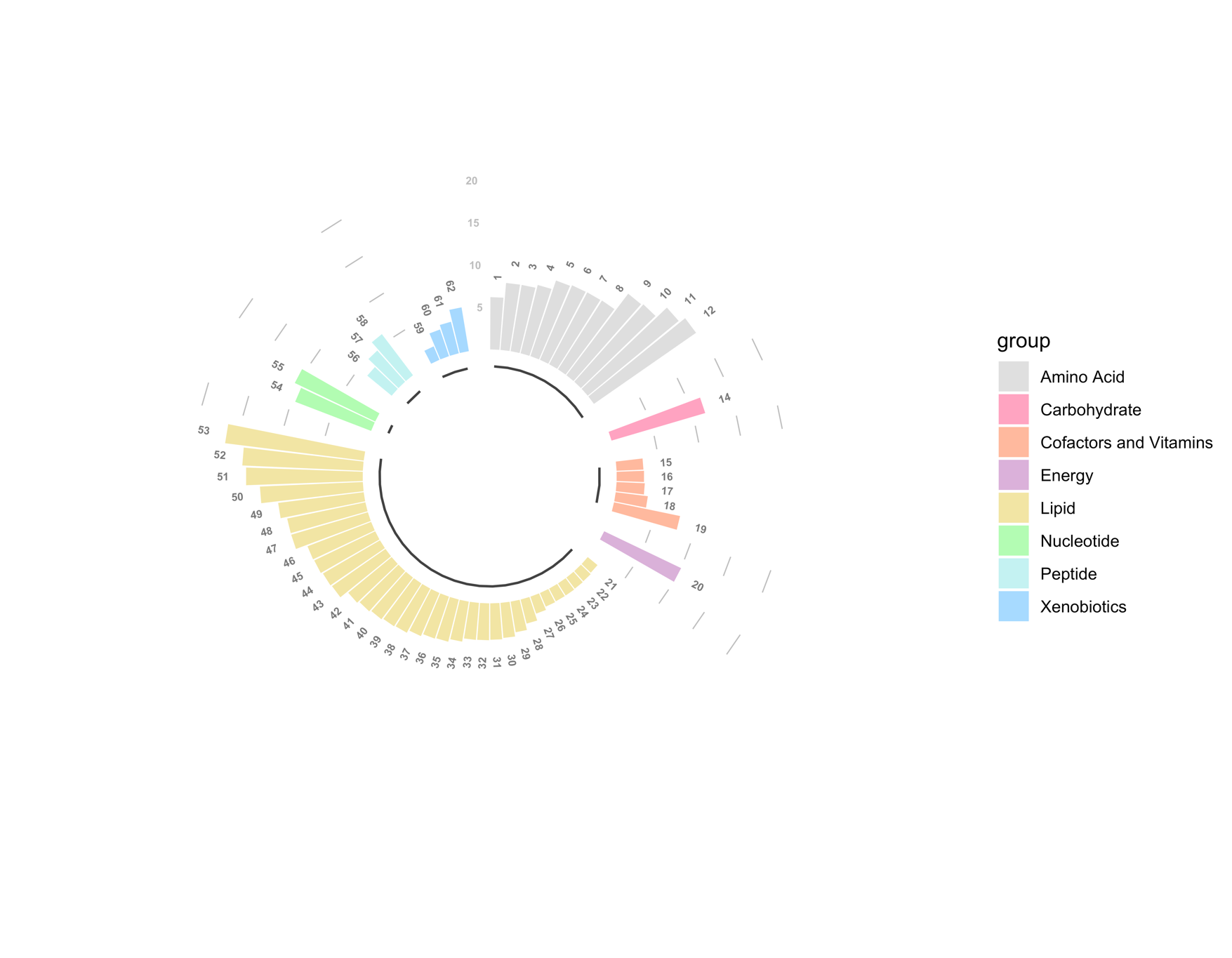

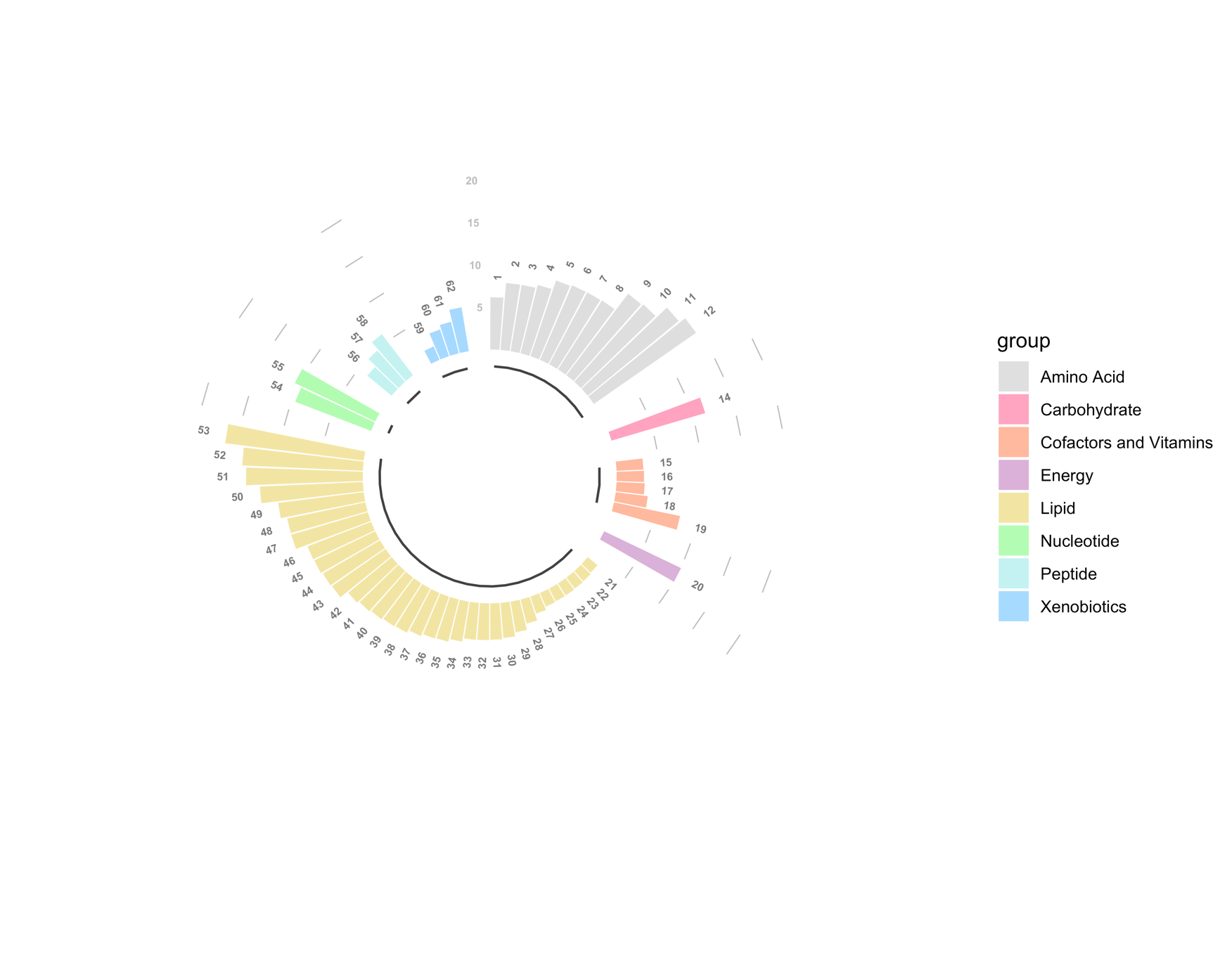
Figure S3.** Scatterplot of standardized mean differences (continuous variables) and absolute difference in proportions (binary variables) comparing the Thai derivation cohort (Ubon-sepsis) and the SE Asian external validation cohort (SEAICRN). All class-defining variables (Table S1) were included. r = Pearson correlation coefficient.

**c**

r = 0.82

P < 0.001

**Figure S4.** Alluvial plot showing the proportion of patients assigned to subphenotype in the original COVID-19 subphenotyping study (CARDS Class 1 and CARDS Class 2) and new classification using the four-variable model (CAP1 and CAP2). *Abbreviations*: ARDS = acute respiratory distress syndrome, CARDS = COVID-19–related ARDS, CAP = community-acquired pneumonia, SOFA = Sequential Organ Failure Assessment.


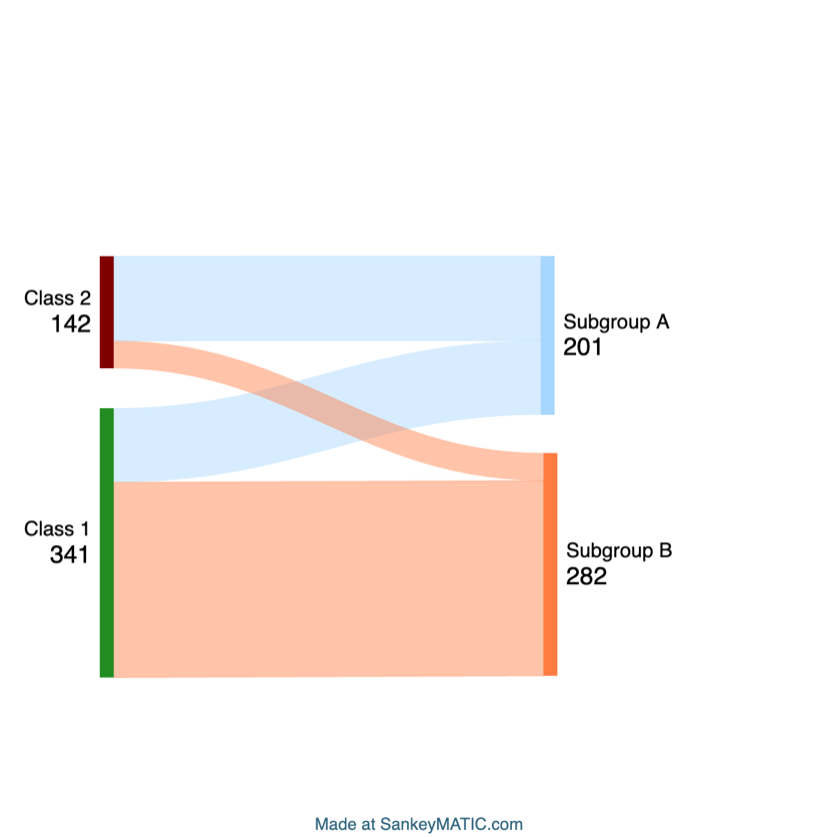


CAP1

CAP2

Class 2

Class 1

48%

12%

23%

30%

**Supplementary References**

1. Hantrakun V, Somayaji R, Teparrukkul P, et al. Clinical epidemiology and outcomes of community acquired infection and sepsis among hospitalized patients in a resource limited setting in Northeast Thailand: A prospective observational study (Ubon-sepsis). *PLoS One*. 2018;13(9):e0204509. doi:10.1371/journal.pone.0204509

2. Southeast Asia Infectious Disease Clinical Research Network. Causes and outcomes of sepsis in southeast Asia: a multinational multicentre cross-sectional study. *Lancet Glob Health*. 2017;5(2):e157-e167. doi:10.1016/S2214-109X(17)30007-4

3. Booraphun S, Hantrakun V, Siriboon S, et al. Effectiveness of a sepsis programme in a resource-limited setting: a retrospective analysis of data of a prospective observational study (Ubon-sepsis). *BMJ Open*. 2021;11(2):e041022. doi:10.1136/bmjopen-2020-041022

4. Pandharipande PP, Shintani AK, Hagerman HE, et al. Derivation and validation of Spo2/Fio2 ratio to impute for Pao2/Fio2 ratio in the respiratory component of the Sequential Organ Failure Assessment score. *Crit Care Med*. 2009;37(4):1317-1321. doi:10.1097/CCM.0b013e31819cefa9

5. Schenck EJ, Hoffman KL, Oromendia C, et al. A Comparative Analysis of the Respiratory Subscore of the Sequential Organ Failure Assessment Scoring System. *Ann Am Thorac Soc*. 18(11):1849-1860. doi:10.1513/AnnalsATS.202004-399OC

6. Al Hussain SK, Kurdi A, Abutheraa N, et al. Validity of Pneumonia Severity Assessment Scores in Africa and South Asia: A Systematic Review and Meta-Analysis. *Healthcare (Basel)*. 2021;9(9):1202. doi:10.3390/healthcare9091202

7. Quinton LJ, Walkey AJ, Mizgerd JP. Integrative Physiology of Pneumonia. *Physiological Reviews*. 2018;98(3):1417-1464. doi:10.1152/physrev.00032.2017

8. Dela Cruz CS, Evans SE, Restrepo MI, et al. Understanding the Host in the Management of Pneumonia. An Official American Thoracic Society Workshop Report. *Ann Am Thorac Soc*. 18(7):1087-1097. doi:10.1513/AnnalsATS.202102-209ST

9. Korkmaz FT, Traber KE. Innate immune responses in pneumonia. *Pneumonia*. 2023;15(1):4. doi:10.1186/s41479-023-00106-8

10. Wittermans E, van der Zee PA, Qi H, et al. Community-acquired pneumonia subgroups and differential response to corticosteroids: a secondary analysis of controlled studies. *ERJ Open Res*. 2022;8(1):00489-02021. doi:10.1183/23120541.00489-2021

11. Sinha P, Meyer NJ, Calfee CS. Biological Phenotyping in Sepsis and Acute Respiratory Distress Syndrome. *Annual Review of Medicine*. 2023;74(1):457-471. doi:10.1146/annurev-med-043021-014005

12. Sinha P, Calfee CS, Delucchi KL. Practitioner’s Guide to Latent Class Analysis: Methodological Considerations and Common Pitfalls. *Crit Care Med*. 2021;49(1):e63-e79. doi:10.1097/CCM.0000000000004710

13. Weller BE, Bowen NK, Faubert SJ. Latent Class Analysis: A Guide to Best Practice. *Journal of Black Psychology*. 2020;46(4):287-311. doi:10.1177/0095798420930932

14. Finch WH, Bronk KC. Conducting Confirmatory Latent Class Analysis Using M *plus*. *Structural Equation Modeling: A Multidisciplinary Journal*. 2011;18(1):132-151. doi:10.1080/10705511.2011.532732

15. Nylund KL, Asparouhov T, Muthén BO. Deciding on the Number of Classes in Latent Class Analysis and Growth Mixture Modeling: A Monte Carlo Simulation Study. *Structural Equation Modeling: A Multidisciplinary Journal*. 2007;14(4):535-569. doi:10.1080/10705510701575396

16. Nguyen HQ, Herting JR, Pike KC, et al. Symptom profiles and inflammatory markers in moderate to severe COPD. *BMC Pulm Med*. 2016;16:173. doi:10.1186/s12890-016-0330-1

17. Wright SW, Lovelace-Macon L, Hantrakun V, et al. sTREM-1 predicts mortality in hospitalized patients with infection in a tropical, middle-income country. *BMC Med*. 2020;18(1):159. doi:10.1186/s12916-020-01627-5

18. Wright SW, Kaewarpai T, Lovelace-Macon L, et al. A 2-Biomarker Model Augments Clinical Prediction of Mortality in Melioidosis. *Clin Infect Dis*. 2021;72(5):821-828. doi:10.1093/cid/ciaa126

19. Xia L, Hantrakun V, Teparrukkul P, et al. Plasma Metabolomics Reveals Distinct Biological and Diagnostic Signatures for Melioidosis. *Am J Respir Crit Care Med*. 2024;209(3):288-298. doi:10.1164/rccm.202207-1349OC

20. Calfee CS, Delucchi K, Parsons PE, et al. Subphenotypes in acute respiratory distress syndrome: latent class analysis of data from two randomised controlled trials. *Lancet Respir Med*. 2014;2(8):611-620. doi:10.1016/S2213-2600(14)70097-9

21. HARRELL Jr. FE, Lee KL, Mark DB. Multivariable Prognostic Models: Issues in Developing Models, Evaluating Assumptions and Adequacy, and Measuring and Reducing Errors. *Statistics in Medicine*. 1996;15(4):361-387. doi:10.1002/(SICI)1097-0258(19960229)15:4<361::AID-SIM168>3.0.CO;2-4

22. Miao Y, Francisco S, Cenzer IS, Kirby KA, Boscardin WJ. Estimating Harrell’s Optimism on Predictive Indices Using Bootstrap Samples. Published online 2013.

23. Ramspek CL, Jager KJ, Dekker FW, Zoccali C, van Diepen M. External validation of prognostic models: what, why, how, when and where? *Clinical Kidney Journal*. 2021;14(1):49-58. doi:10.1093/ckj/sfaa188

24. Noma H, Shinozaki T, Iba K, Teramukai S, Furukawa TA. Confidence intervals of prediction accuracy measures for multivariable prediction models based on the bootstrap-based optimism correction methods. *Statistics in Medicine*. 2021;40(26):5691-5701. doi:10.1002/sim.9148

25. Ramachandran KM, Tsokos CP. Chapter 13 - Empirical methods. In: Ramachandran KM, Tsokos CP, eds. *Mathematical Statistics with Applications in R (Third Edition)*. Academic Press; 2021:531-568. doi:10.1016/B978-0-12-817815-7.00013-0

26. Hosmer DW, Hosmer T, Le Cessie S, Lemeshow S. A comparison of goodness-of-fit tests for the logistic regression model. *Stat Med*. 1997;16(9):965-980. doi:10.1002/(sici)1097-0258(19970515)16:9<965::aid-sim509>3.0.co;2-o

27. Sinha P, Furfaro D, Cummings MJ, et al. Latent Class Analysis Reveals COVID-19-related Acute Respiratory Distress Syndrome Subgroups with Differential Responses to Corticosteroids. *Am J Respir Crit Care Med*. 2021;204(11):1274-1285. doi:10.1164/rccm.202105-1302OC
